# Model-based analysis to identify the impact of factors affecting electricity gaps during COVID-19: A case study in Germany

**DOI:** 10.1016/j.heliyon.2024.e33943

**Published:** 2024-07-03

**Authors:** Nanae Kaneko, Yu Fujimoto, Hans-Arno Jacobsen, Yasuhiro Hayashi

**Affiliations:** a School of Advanced Science and Engineering, Waseda University, Tokyo, Japan; b Advanced Collaborative Research Organization for Smart Society, Waseda University, Tokyo, Japan; c Department of Electrical & Computer Engineering, University of Toronto, Canada

**Keywords:** ARIMAX, COVID-19, Electricity demand, Factor analysis, Machine learning, Sparse modeling

## Abstract

The recent COVID-19 pandemic has precipitated drastic changes in economic and lifestyle conditions, significantly altering residual electricity demand behavior. This alteration has expanded the demand gap between actual and forecasted electricity usage based on pre-pandemic data, highlighting a critical global issue. Many studies in the pandemic have explored the features of this widening gap, which is impacted by major social events like fast virus spread and lockdowns. However, the influence of factors like economic shifts and lifestyle changes on this demand remains largely unexplored, primarily due to the pandemic's significant effects in these areas. Understanding the essential factors affecting the demand gap is crucial for stakeholders in the electricity sector to develop effective strategies. This study examines the hourly electricity consumption and related factors during the specified period. We present a method combining time-series forecasting and sparse modeling. This helps identify critical factors affecting the electricity demand gap during the pandemic, highlighting the most crucial variables. Utilizing this method, we identify the variables that have undergone significant changes during the pandemic and evaluate their effects on the electricity demand gap. The effectiveness is proven by applying it to the dataset collected in German.

## Highlights

• COVID-19 pandemic has drastically transformed economic and lifestyle conditions.
• Electricity demand gap between actual and forecasted demands widened post-pandemic.
• The proposed approach evaluated the demand gap caused by the pandemic.
• Partially linear additive model identified the impact of key factors on demand gap.
• A case study is conducted for Germany to validate the proposed approach.

**Table:** Symbols

T	Hourly data training period
T	Number of samples per hour
D	Daily data training sessions
D	Number of samples per day
M	Monthly data training period
M	Number of samples per month
Y	Yearly data training period
Y	Number of samples per year
h	Index for denoting a particular day
d	Index for denoting a particular day
m	Index for denoting a particular month
y	Index for denoting a particular year
$x_{d}^{j}$	Observation of explanatory variables on day d
$x_{m}^{j}$	Observation of explanatory variables on month m
$x_{y}^{j}$	Observation of explanatory variables on year y
$h_{a}\left(d\right)$	Indicator function to derive the dummy variables based on the month
$h_{b}\left(d\right)$	Indicator function to derive the dummy variables based on the day of the week
κ	Coefficient parameters for the autoregressive component
μ	Coefficient parameters for the moving average component
ν,ξ	Coefficient parameters for the exogenous variables
$l_{ymdh}$	Hourly demand recorded at hour h on day d in month m of year y
J	Number of explanatory variables
$x_{ymdh}$	$=\left(x_{ymdh}^{1},\ldots ,x_{ymdh}^{J}\right)\text{.}$ Explanatory variables observed at hour h on day d in month m of year y
L	⊆S. Index subset showing variables linearly linked to target demand
N	⊆S. Index subset showing variables nonlinearly linked to target demand
β	Coefficient parameters for explanatory variables
$\varphi_{j}^{k}\left(.\right)$	Function for cubic spline transformation
K	Number of bases in cubic spline transformation functions
$\tau_{j}$	Vector of coefficient parameters for transformation functions $\varphi_{j}^{1}\left(x^{j}\right),\ldots ,\varphi_{j}^{K}\left(x^{j}\right)$
λ	Positive constant penalty for regularization
S	={1,…,P}. Index set of explanatory variables
$S_{mh}^{\left(i\right)}$	Index subset of the selected variables with nonzero components
$Gap_{ymdh}$	Electricity demand deviation at hour h on day d in month m of year y
${\hat{S}}_{\;mh}^{\text{deviance}}$	Index of the critical variables deviating from the scenarios without COVID-19 in each seasonal period
${\hat{S}}_{\;mh}^{\text{expected}}$	Variable index varying within the expected scenario
Nomenclature	
GDP	Gross domestic product
ARIMAX	Autoregressive integrated moving average with an exogenous variable
PLAMs	Partially linear additive models
HICP	Harmonized Indices of Consumer Prices
RMSE	Root mean squared errors

## 1 Introduction

### 1.1 Background

After the COVID-19 outbreak, economic changes and shifts in consumer habits have impacted electricity consumption patterns [1,2]. Moreover, recent developments in electricity power systems, such as the widespread adoption of household-scale power sources like solar energy and increased interest in energy efficiency, have intensified these effects on residual electricity demand. This term indicates the difference between overall national electricity usage and the portion derived from renewable sources [3]. Addressing these demand fluctuations presents a global challenge; discrepancies between actual demand during the pandemic (COVID-19 demand scenario) and projections based on pre-pandemic data (non-COVID-19 demand scenario) have introduced uncertainties into decision-making processes for electricity utilities, impacting areas like reserve planning and facility design. These shifts in demand patterns could affect the energy economy and progress towards global greenhouse gas reduction targets [4]. Therefore, stakeholders in the electricity sector must grasp the factors influencing these demand gaps to formulate effective strategies [5].

### 1.2 Related works

Historically, many studies have investigated electricity demand, suggesting various approaches to analyzing the statistical connections among variables that depict consumption patterns [[6], [7], [8]]. Initially, early studies emphasized a limited set of variables determined by practical experience. For instance, Hor et al. [6] analyzed the correlation between monthly electricity demand and elements like weather and gross domestic product (GDP). With the evolution of the electricity system, demand may now be influenced by extra factors such as energy-saving practices and the costs of essential goods [9,10].

Lockdown measures and social distancing policies during the COVID-19 pandemic led to substantial decreases in electricity demand. In China, Huang et al. [1] found that electricity demand varied by 12 % during COVID-19 compared to regular times, correlating with lockdown stringency. Similarly, Chen et al. [11] investigated how electricity demand correlates with consumer mobility data, affirming that variations in lockdown severity drove the substantial discrepancies between the COVID-19 and non-COVID-19 demand scenarios. Such analyses, which focus on lockdown severity data, provide a rapid means to assess the pandemic's impact on electricity demand. Additionally, the impact of the demand gap during the pandemic may differ depending on the types of commercial, industrial, and residential activities within the specific area [12]. The pandemic's impact on particular sectors may lead to significant, unforeseen fluctuations in electricity demand. Exploring the impact of COVID-19 on electricity demand from various sectors like commercial, industrial, and residential activities remains uncharted, mainly in academic research.

### 1.3 Contributions

This study aims to employ a statistical model-based approach to analyze the effects of commercial, industrial, and residential activities on the electricity demand gap between the COVID-19 and non-COVID-19 demand scenarios. Conventional methods typically involve aligning time-series data on electricity demand gaps with various variables to identify correlations visually. These methods facilitate an intuitive understanding of data relationships. In contrast, developing a statistical regression model that identifies key variables from a dataset enables a quantitative analysis of how variations in each variable specifically impact the electricity demand gap. This approach offers a more comprehensive assessment than simple data alignment, providing deeper insights into the dynamics influencing electricity consumption during the pandemic.

The research questions (RQ) for this study are outlined as follows:

RQ1 What are the significant commercial, industrial, and residential changes during the COVID-19 pandemic?

RQ2 Which key activities significantly impact electricity demand?

RQ3 How do these key activities quantitatively affect the gap in electricity demand between the COVID-19 and non-COVID-19 demand scenarios?

This study focuses on the hourly residual electricity demand, defined as the total electricity demand minus the supply from renewable sources. We consider a broad array of potential explanatory variables, including aspects of power systems, economic conditions, and consumer interests, which previous studies have not sufficiently addressed. We aim to identify the key factors defining the hourly electricity gap induced during the COVID-19 crisis. We propose a data-driven methodology utilizing autoregressive integrated moving average with an exogenous variable (ARIMAX) [13] and enumerated sparse partially linear additive models (enumerated sparse PLAMs) [10] to explore these questions. ARIMAX is commonly used for forecasting time-dependent data by incorporating external or exogenous variables and is employed here to simulate scenarios under non-COVID-19 conditions. The enumerated sparse PLAM approach is crucial for pinpointing a limited number of key variables that define the situation-dependent hourly electricity demand, enabling us to analyze changes in the informative variables effectively. This study uncovers how key factors affect the electricity demand gap amid the pandemic, offering valuable insights for electricity sector stakeholders.

This paper's main contributions include:

1. We thoroughly analyze how key variables affect the electricity demand gap, including several potential variables that have not been extensively studied in demand gap modeling literature.
2. We employ ARIMAX to identify deviations in explanatory factors between the COVID-19 period and a non-COVID-19 scenario, estimated using pre-pandemic data.
3. We implement enumerated sparse PLAMs for demand modeling and determine key factors influencing demand gaps.
4. We propose a methodology to quantify the impact of each key factor by comparing the actual COVID-19 demand scenario with a hypothetical non-COVID-19 scenario derived from the constructed PLAMs.
5. Utilizing this framework, we analyze a real-world dataset to explore the key factors that impact hourly electricity consumption.

### 1.4 Organization of the paper

This paper is organized as follows: Section 2 reviews the electricity demand gap analysis and outlines the features of the specific electricity demand and several explanatory factors. Section 3 outlines the proposed methodology for identifying essential factors affecting pandemic-era demand and their impact on the electricity demand gap. Section 4 examines the outcomes of using the proposed framework on a German dataset, a country heavily affected by the pandemic [14]. It offers information on how the identified variables affect the electricity demand gap. Section 5 concludes the findings of this study.

## 2 Identification of important factors characterizing the electricity demand gap

### 2.1 Literature review

Table 1 summarizes previous studies on the behavior of electricity demand. Traditionally, analyses have predominantly relied on a small number of factors like weather and economic conditions to understand how they impact the target demand. However, in cases where power demand exhibits complex variations, the analysis incorporating a broader array of variables. For instance, Kaneko et al. [10] examined the hourly electricity demand in Japan, identifying critical variables that influence changes in demand by analyzing numerous factors, including weather, interest rates, stock prices, calendar effects, and GDP data. Post-COVID-19, several studies have examined the pandemic's impact on electricity demand changes; the effects of lockdown scale and infection rates were primarily emphasized. Ceylan et al. [15] investigated the daily electricity consumption patterns and analyzed how lockdown measures affected demand changes. Similarly, Chen et al. [11] developed a method to model daily demand, incorporating mobility data to indicate economic activity. These studies suggest that the pandemic has significantly impacted the electricity demand gap between the COVID-19 scenario and the non-COVID-19 scenario, with these effects varying based on factors such as population densities, economic development, political orientations, and COVID-19 management strategies. Moreover, Alavi et al. [12] noted that the demand gap tends to be more pronounced in commercial and industrial areas compared to residential areas. However, prior related research has not thoroughly explored the relationships between specific business categories and the demand gap.

**Table 1:** Representative prior studies on variations in electricity demand.

Study	Target	Factors	Region
Vu et al. (2015) [16]	Peak monthly demand	Weather	Australia
Matsukawa (2016) [17]	Daily peak demand	Weather, electricity prices	Japan
Huang et al. (2016) [18] Kumar et al. (2018) [19]	Hourly demand	Weather	China, India
Honjo et al. (2018) [5]	Monthly peak demand	Weather, the economic production index, GDP, electricity price indices, real wage index, population	Japan
Zhang et al. (2019) [8]	Peak daily demand	Weather, Income	China
Corinaldesi et al. (2019) [3]	Annual peak demand	PV generation	Europe
Kaneko et al. (2020) [10]	Hourly demand	Weather, economic index, GDP, price index, Internet searches index, Calendar	Japan
Chen et al. (2020) [11]	Daily demand	Mobility data	UK, Germany, France
Huang et al. (2021) [1]	Monthly demand	Number of COVID-19 cases	China
Ceylan et al. (2021) [15]	Monthly demand	Curfew, Temperature, Holidays	Turkey
Navon et al. (2021) [20]	Daily average demand	Lock down during COVID-19 pandemic	France, Spain
Hauser et al. (2021) [21]	Weekly average demand	Lock down during COVID-19 pandemic	Germany, U.K.
Celik et al. (2022) [2]	Annual monthly demand	Cooling/heating degree days, Renewable energy output	China, U.K., France, Spain, Germany, India, Italy
Li et al. (2022) [22]	Daily demand	Effective reproduction numbers during COVID-19 pandemic	U.S., Germany
Alavi et al. (2022) [12]	Daily/monthly average demand	Lockdown, Cyclone	Bangladesh
Dai et al. (2023) [23]	Hourly demand	Poverty, income level, and race data	U.S.

### 2.2 Empirical studies of behavior of electricity demand in Germany

**Fig. 1:**
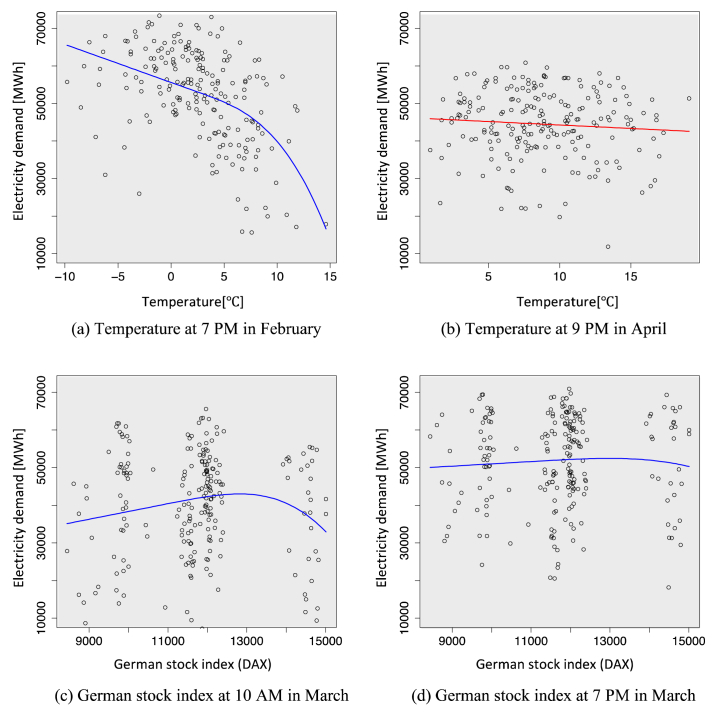
Relationships between seasonal electricity demand and several factors, with solid lines representing curves derived from the sparse PLAM (refer to Section 3). These factors include: (a) Temperature at 7 p.m. in February, (b) Temperature at 9 p.m. in April, (c) German stock index at 10 a.m. in March, and (d) German stock index at 7 p.m. in March.

Various factors, such as weather, renewable energy capacity, economy, and conservation habits, can affect electricity demand (see Table 2 for details). This study specifically focuses on the hourly electricity demand in Germany [24], a region that has experienced significant economic and social repercussions due to the pandemic, which has also profoundly impacted the gap in electricity demand. Fig. 1(a)–(d) show how electricity demand relates to different factors in specific seasons; solid lines represent relationships identified by the sparse PLAM introduced in Section 3. The figure highlights how electricity demand fluctuates in response to these factors. Notably, the relationships between the variables are not consistently linear and may change based on seasonal conditions. Therefore, our study focuses on the linear/nonlinear behavior of the hourly electricity consumption curve.

**Table 2:** Categories of explanatory variables used.

Attribute: Number of the variables	
Installed generation capacity [24]: v1-12	Yearly
Weather [37]: v12-17	Hourly
DAX [26]: v18	Daily
Indices of production in industry [25]: v19-47	Monthly
Indices of production in service [38]: v48-70	
Indices of production in construction [39]: v71-72	
GDP [40]: v73	
Producer price index [41]: v74-167	
Number of internet searches (power-related words) [42]: v168-171	
Calendar data (holiday/weekday/day of week; binary dummy): v172-179	Daily

* All explanatory variables are standardized to have zero means and unit variances.

**Fig. 2:**
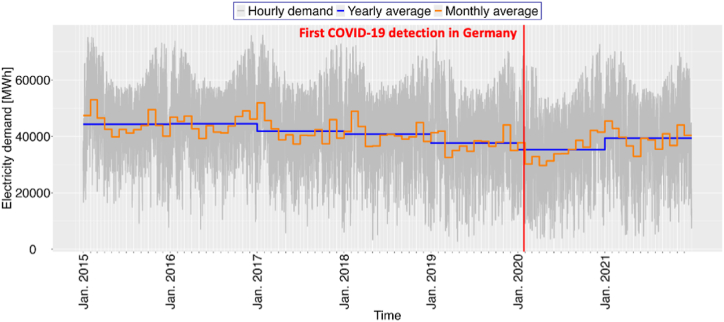
Behavior of electricity demand. The red line indicates the date COVID-19 was first detected, the blue line represents the annual average electricity demand, and the orange line represents the monthly average electricity demand. (For interpretation of the references to color in this figure legend, the reader is referred to the Web version of this article.)

Since the onset of the COVID-19 pandemic in 2020, political interventions such as lockdowns and shutdowns have drastically altered economic conditions, pricing structures, and electricity consumption behaviors, leading to considerable fluctuations in electricity demand. Fig. 2 shows the monthly and yearly electricity demand from 2015 to 2021. The annual electricity demand in Germany has been slowly decreasing, possibly because of the rise in small household-scale power sources like rooftop solar installations. Despite a decrease in average electricity demand in 2020, there was a noticeable increase in 2021 as the pandemic's immediate impacts began to wane.

**Fig. 3:**
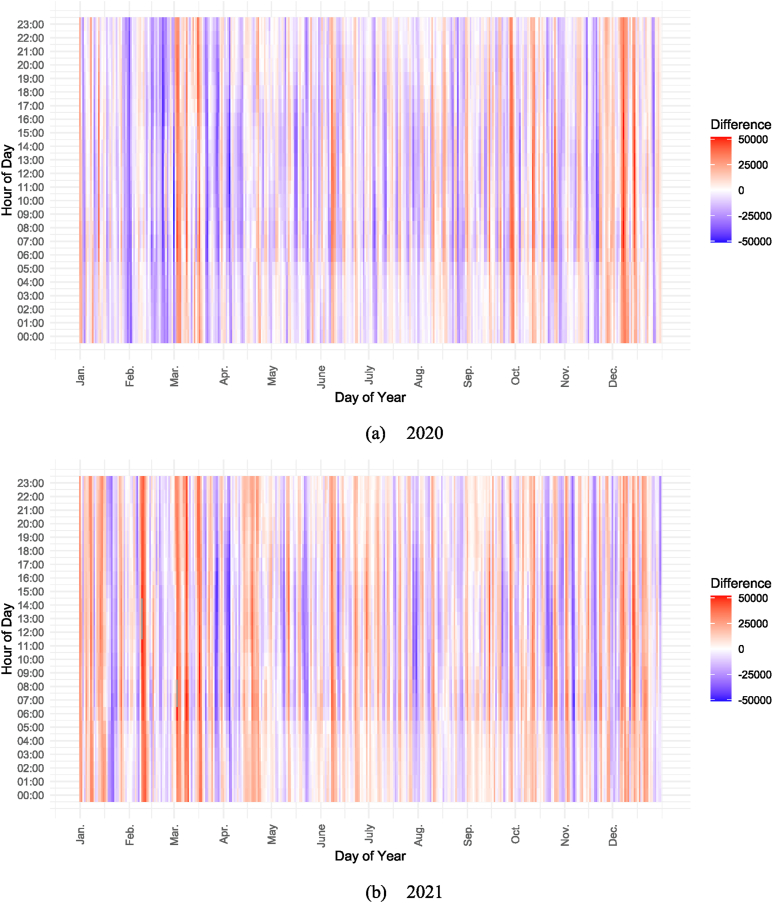
Difference between the electricity demand (a) in 2020 and (b) in 2021 and the electricity demand in 2019.

Fig. 3(a) and (b) show the difference between the electricity demand in 2020 and 2021 and the electricity demand in 2019. The figures illustrate the variations in electricity demand compared to 2019 under different circumstances: in 2020, there was a general decrease in electricity demand compared to 2019, although there were intervals where demand exceeded that of the previous year. Conversely, in 2021, there tended to be extended periods where electricity demand exceeded the levels recorded in 2019.

**Fig. 4:**
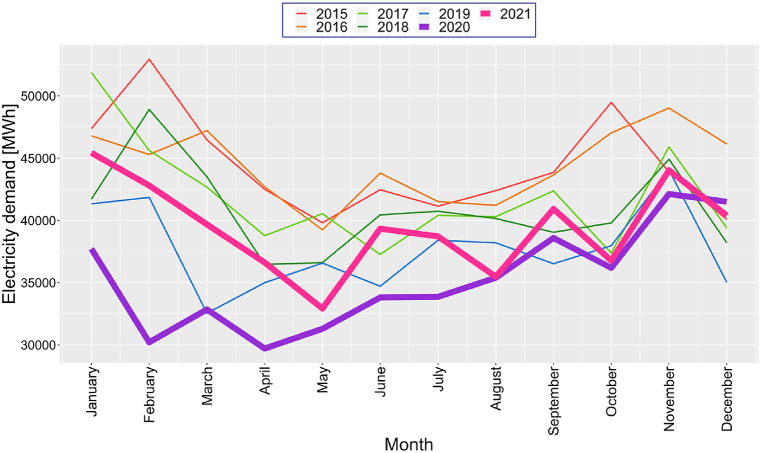
Annual monthly electricity demand from 2015 to 2021.

**Fig. 5:**
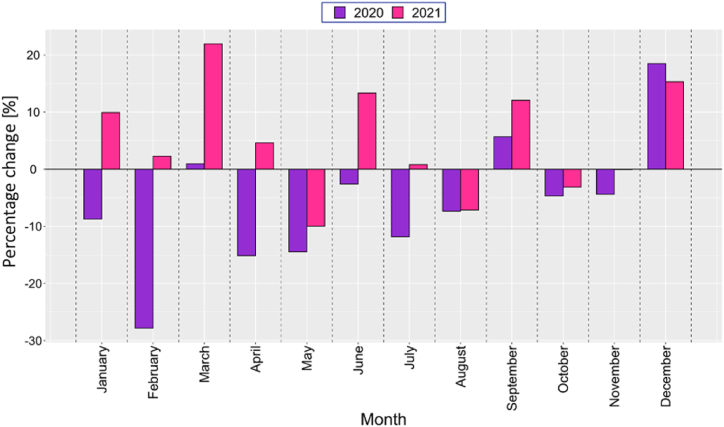
Comparative analysis of monthly electricity demand from 2020 to 2021 against the same period in 2019.

Fig. 4 shows the monthly average electricity demand changes from 2015 to 2021, while Fig. 5 compares demand variations in 2020 and 2021 with 2019. These figures suggest that, in April 2020, electricity demand decreased significantly by 27.8 %, followed by a strong recovery in March 2021 with a 21.9 % surge compared to 2019. This analysis underscores the dynamic nature of electricity demand under varying economic and social conditions.

**Fig. 6:**
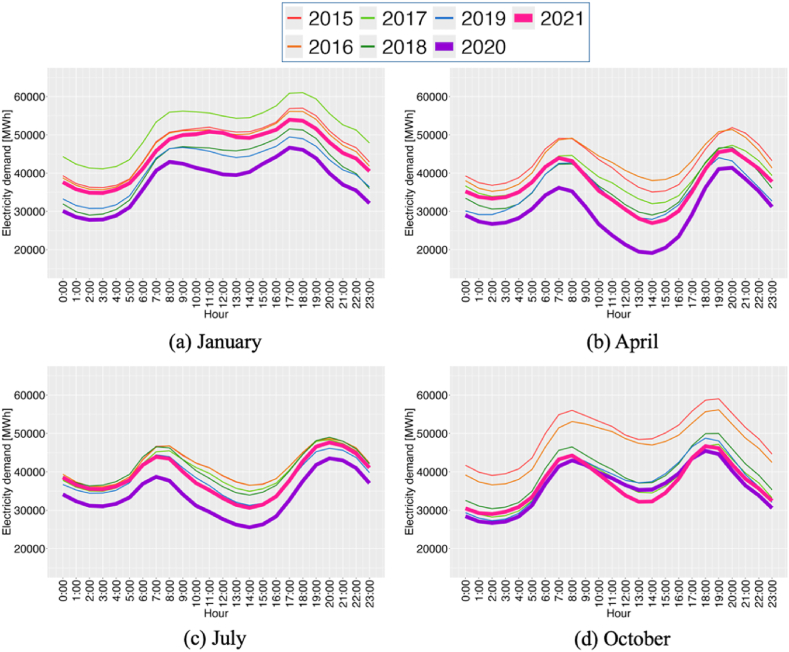
Sample averages of hourly electricity demand (a) in January, (b) in April, (c) in July and (d) in October.

The average daily demand patterns were analyzed for different months from 2015 to 2016, shown in Fig. 6(a)-(d). These patterns vary in timing and intensity across seasons. In Germany, electricity usage typically decreases during the day, especially in 2020 and 2021, reflecting pandemic-related changes affected by seasons and specific time slots.

**Fig. 7:**
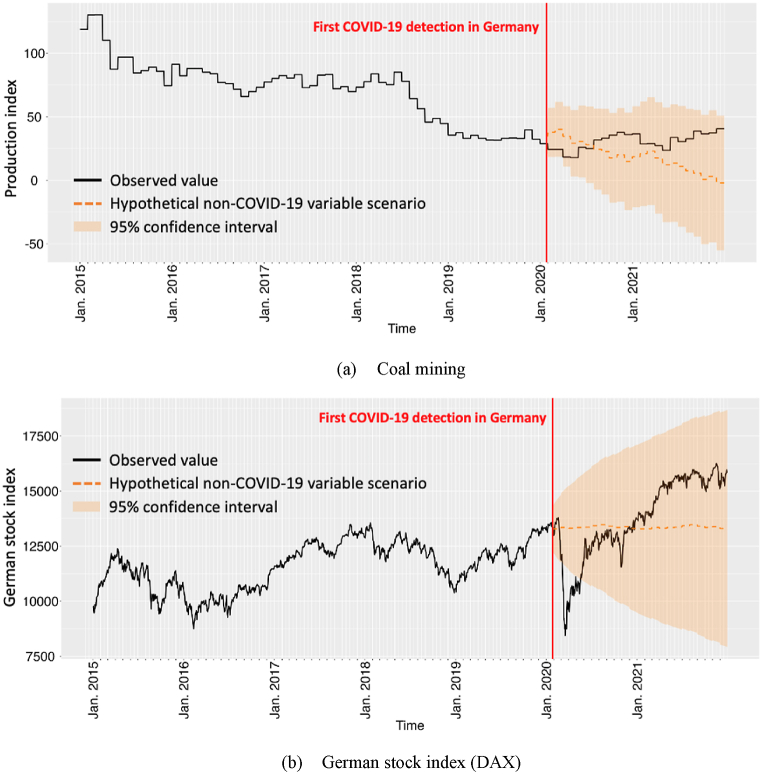
Dynamics of explanatory variables across two scenarios: pre-pandemic and during the pandemic. Specifically, it focuses on: (a) Coal mining and (b) the German stock index (DAX). The black line represents the observed variable, while the orange line and shaded area depict the non-COVID-19 scenario and the 95 % confidence interval, respectively. This scenario is derived using pre-pandemic data through the ARIMAX method, as explained in Section 3.1. (For interpretation of the references to color in this figure legend, the reader is referred to the Web version of this article.)

Fig. 7 presents the long-term trends of several critical variables influencing electricity demand from 2015 to 2021. For instance, Fig. 7(a) displays the production index^1^ of the coal mining industry [25], where certain variables remained stable over the period. However, the trends of several variables shifted during the pandemic. The behavior of the German stock index [26], as shown in Fig. 7(b), reflects the domestic economic conditions, which deteriorated significantly during the pandemic. The filled ranges in these figures represent the expected variations for 2020–2021, using data trends up to 2019. The data show notable shifts in the behaviors of these variables that changed significantly during the pandemic; the restrictions and lockdowns substantially impacted economic conditions and consumer habits.

### 2.3 Theoretical background of analysis of electricity demand

Many researchers and operators have tried to develop data-driven models for studying electricity demand. Most existing studies have relied on a narrow range of variables based on experts' empirical knowledge. They concentrate on factors affecting demand, like economic indicators such as GDP and national holidays [6,7]. Additional factors like lockdown severity have also been incorporated post-pandemic, as shown in Table 2. However, given the complexity of power systems, more comprehensive analyses that consider a wider range of variables—including demographic, climatic, commercial, industrial, and residential factors—have been discussed [5,10]. These studies indicate that the main factors affecting temporal variations in electricity demand may vary depending on the particular situation and target dynamics. High numbers of explanatory variables lead to unstable estimation results due to multicollinearity [27]. Some variables, including redundancies, inadequately capture changes in electricity demand. The concept of sparse modeling in machine learning focuses on choosing crucial variables and pinpointing those with low relevance to the target value [28]. This method has successfully solved various complex, real-world, ill-defined problems. We can understand how factors impact different situations by constructing models using data from particular seasons, pinpointing critical explanatory factors, and comparing them with models from other conditions.

Moreover, while previous studies like Dai et al. [23] have assumed simple linear relationships between the demand gap and influencing factors, utilizing statistical models based on these assumptions to analyze the main impacts of each factor on demand, recent research has increasingly highlighted the importance of considering nonlinear relationships. For instance, Huang et al. [18] presented a method using nonparametric and nonlinear modeling to enhance the description of target demand. However, such nonlinear approaches, which consider the complex effects of interactions between variables, often make it challenging to isolate the main effects of individual variables on the target value. In this context, PLAM effectively separates linear and nonlinear main impacts of explanatory variables [29], excluding complex interactions.

In the next section, we introduce a scheme utilizing sparse PLAMs. This approach is highly promising for describing the behavior of electricity demand and identifying key variables that influence the target demand in both linear and nonlinear ways.

## 3 Analysis of the gap in electricity demand during pandemic

### 3.1 Overview of the analysis of the gap in electricity demand

**Fig. 8:**
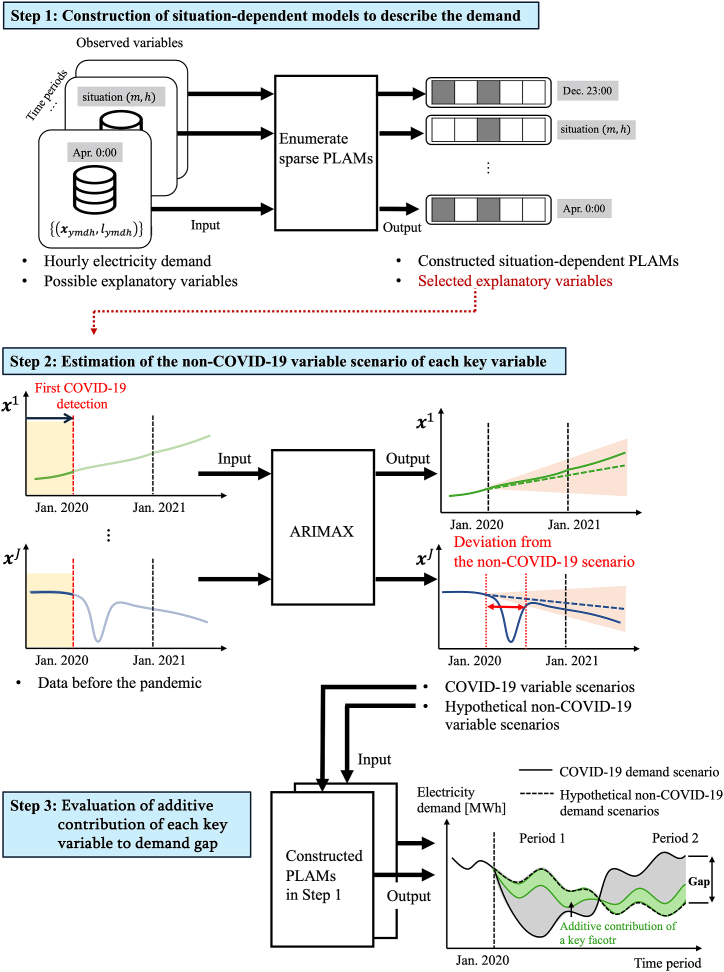
Analysis of the electricity demand gap. This figure demonstrates how COVID-19/non-COVID-19 demand scenarios are derived using enumerated sparse PLAMs and ARIMAX.

We focus on the hourly residual electricity demand and propose an approach to select key variables that describe this demand, as well as to identify the additive contribution of each variable to the electricity demand gap between the COVID-19 demand scenario and a non-COVID-19 scenario, which is hypothetically estimated using pre-pandemic data. Fig. 8 shows an overview of the proposed approach.

We employed a dataset spanning from 2015 to 2021, which includes actual residual demand and hundreds of potential explanatory variables. These variables are grouped into categories such as weather, stock prices, commodity prices, and calendar data (see Table A1 in the Appendix for more details). In Step 1, we used the enumerated sparse PLAM to select key variables for explaining electricity demand. This model is particularly effective for identifying a limited number of critical variables that elucidate the situation-dependent behavior of annual demands from numerous candidates. Additionally, it determines whether the plausible relationships between the selected variables and the target demand are linear or nonlinear [10]. In Step 2, we employ the ARIMAX model to estimate the hypothetical non-COVID-19 variable and demand scenarios using pre-pandemic data. The ARIMAX model is an extension of the ARIMA model, widely recognized for its efficacy in describing relationships between target demand and historical data. ARIMAX enhances this approach by incorporating exogenous explanatory variables, allowing for a more comprehensive modeling of demand behavior under different scenarios [[30], [31], [32]]. In Step 3, we estimate the gap in electricity demand between the COVID-19 scenario and the hypothetical non-COVID-19 scenario. This estimation is based on the constructed situation-dependent demand models and the variable scenarios for both COVID-19 and non-COVID-19 conditions. Here, COVID-19 and non-COVID-19 demand scenarios are defined and calculated as follows.

• The COVID-19 demand scenario is a time series of data showing the behavior of electricity demand under actual pandemic conditions; it is described by introducing the COVID-19 variable scenarios (i.e., observed data) into the PLAMs constructed in Step 1.
• The non-COVID-19 demand scenario is time series data of hypothetical demand assumed in the absence of a pandemic; it is derived by introducing the estimated non-COVID-19 variable into the PLAMs constructed in Step 1.

During the pandemic, several variables exhibited unexpected and significant changes, while others varied in predictable ways in response to economic fluctuations. In this study, we propose an approach to identify the key variables that deviate significantly during the pandemic based on estimated confidence intervals around the non-COVID-19 variable scenarios and to determine the additive contributions of these variables to the deviance-oriented gap. As a whole procedure, we analyze the impact of the critical variables related to the changes in the electricity demand during the pandemic.

### 3.2 Situation-dependent modeling based on partially linear additive models

In Step 1, we developed a situation-dependent model to describe the hourly electricity demand. Initially, we targeted specific demands and corresponding explanatory variables for each period to create models that accurately represent the behavior of hourly demand during these periods. This approach essentially illustrates how hourly electricity demand is influenced by various time-related factors.

Let $S=\left\{1,\ldots ,J\right\}$ be an index subset of explanatory variables, where $\left\{\left(x_{ymdh},l_{ymdh}\right)\right\}$ is a set of pairs containing $l_{ymdh}$, the observed electricity demand at hour h on day d in month m of year y; and $x_{ymdh}=\left(x_{ymdh}^{1},\ldots ,x_{ymdh}^{J}\right)$ is a vector of J variables observed at the corresponding timing. The daily, monthly, and yearly variables were interpolated using the nearest-neighbor method. Moreover, we let L⊆S and N⊆S be the index subsets of $S=\left\{1,\ldots ,J\right\}$ to indicate the linear and nonlinear variables related to the target demand. The PLAM provides a formulation for describing the target demand using various variables with both linear and nonlinear relationships, defined in Eq. (1) as follows:

$$l_{ymdh}≅f\left(x_{ymdh};\theta \right)$$

(1) $$=\beta_{0}+\sum_{j\in S}\left(\left(\beta_{j}+\tau_{j,1}\;\right)x_{ymdh}^{j}+\sum_{k=2}^{K}\tau_{j,k}\varphi_{j}^{k}\left(x_{ymdh}^{j}\right)\right)\text{,}$$

where $\theta =\left\{\beta =\left({\beta_{0},\beta }_{1},\ldots ,\beta_{J}\right),\left\{\tau_{j}=\left\{\tau_{j,1},\ldots ,\tau_{j,K}\right\}\right\}\right\}$ indicates a set of model coefficient parameters, β denotes a set of the coefficient parameters for the explanatory variables x, and $\tau_{j}$ represents the vector of coefficient parameters for transformation functions $\varphi_{j}^{1}\left(x^{j}\right),\ldots ,\varphi_{j}^{K}\left(x^{j}\right)$. Additionally, we employ cubic spline transformation functions as Eqs. (2), (3), known for their effectiveness with the PLAM in prior research [10], with K bases:

(2) $$\left[\varphi_{j}^{k}\left(x_{ymdh}^{j}\right);k=1,..,K\right]=\left[x_{ymdh}^{j},{\left(x_{ymdh}^{j}-x_{\left(1\right)}^{j}\right)}_{+}^{3},\ldots ,{\left(x_{ymdh}^{j}-x_{\left(K-1\right)}^{j}\right)}_{+}^{3}\right]\text{,}$$

(3) $${\left(z\right)}_{+}=\left\{ \begin{array}{c} 0\;\left(z<0\right)\\ z\;\left(z\geq 0\right) \end{array}\right.\text{,}$$

where $x_{\left(1\right)}^{j},\ldots ,x_{\left(K-1\right)}^{j}$ represent the knots of the spline selected from quantiles in the sample set.

The sparse modeling technique systematically selects informative variables, determining linear or nonlinear relationships between explanatory variables and target demand. For a specific month m and hour h, the parameters can be estimated based on minimizing the squared error loss $F_{mh}$ under the given subsets L and N, defined in Eq. (4) as follows:

(4) $${\hat{\theta }}_{mh}=\underset{\theta }{\mathrm{argmin}}F_{mh}\left(\theta ;S,\lambda \right)=\underset{\theta }{\mathrm{argmin}}\sum_{y,d}{\left(l_{ymdh}-f\left(x_{ymdh};\theta \right)\right)}^{2}+\lambda \sum_{j\in S}\left(\left|\beta_{j}\right|+{\left\|\tau_{j}\right\|}_{2}\right)\;\text{,}$$

where λ denotes a positive regularization constant for the penalty, and ${\left\|\tau \right\|}_{2}=\sqrt{\sum_{k=1}^{K}\tau_{k}^{2}}$ represents the L2-norm of the vector τ. The parameter λ influences the modeling outcomes and is determined based on a one-day walk-forward validation [33]. The components of the minimizer $\hat{\theta }$ in Eq. (4) approach zero to minimize absolute and L2-norm penalties, along with decreasing the squared error loss. This effect leads to redundant variables approaching zero, making it easier to select a subset of explanatory variables with multiple informative variables [29]. In Eq. (4), the regularization process identifies essential variables based on seasonal conditions and determines their linear or nonlinear relationships as follows:

• $\beta_{j}\neq 0,\tau_{j,k}=0\;\left(\forall k\right)$: Variable $x^{j}$ exhibits a linear relationship with demand.
• $\tau_{j,k}\neq 0\;\left(\exists k\right)$: Variable $x^{j}$ exhibits a nonlinear relationship with demand.
• $\beta_{j}=0,\tau_{j,k}=0\;\left(\forall k\right)$: Variable $x^{j}$ exhibits no relationship with demand.

To enhance the interpretability of variables that significantly influence electricity demand on an annual basis, the enumerated sparse modeling technique [34] is utilized. This technique mechanically selects a limited number of consistently relevant variables across various situational models. The enumeration scheme builds models by choosing vital variables and assessing their linearity or nonlinearity. In this scheme, plausible candidate models are enumerated based on the specific situation (m,h), and a representative model is chosen from these candidates to minimize the overlap of explanatory variables commonly used in models tailored for individual situations {(m,h)}. Let $\left(S_{mh}^{\left(1\right)},F_{mh}^{\left(1\right)}\right)$ be a set of pairs containing the index set of variables selected by the sparse PLAM as expressed in Eq. (1) and the squared-error loss under that variable set $S_{mh}^{\left(1\right)}$, and let $\left\{\left(S_{mh}^{\left(2\right)},\ldots ,S_{mh}^{\left(I_{mh}\right)}\right),\left(F_{mh}^{\left(2\right)},\ldots ,F_{mh}^{\left(I_{mh}\right)}\right)\right\}$ be the set of variable indices and the corresponding squared error losses enumerated in the sparse PLAM scheme. These enumerated sets of variables $\left(S_{mh}^{\left(2\right)},\ldots ,S_{mh}^{\left(I_{mh}\right)}\right)$ contain almost identical information to describe electricity demand, and the sets of squared-error loss $\left(F_{mh}^{\left(2\right)},\ldots ,F_{mh}^{\left(I_{mh}\right)}\right)$ exhibit only minor differences. Therefore, the squared error loss in every enumerated model satisfies the following requirements in Eq. (5):

(5) $$0<\frac{F_{mh}^{\left(i\right)}-F_{mh}^{\left(1\right)}}{F_{mh}^{\left(1\right)}}<\varepsilon \;\left(\forall i\in \left(2,\ldots ,I_{mh}\right)\right)\text{,}$$

where the positive parameter ε regulates the suboptimality of the enumerated results. Specifically, the situation-dependent key variables are selected based on these criteria in Eq. (6).

(6) $$\hat{S}=\min_{\left\{i_{mh}\in \left\{1,\ldots ,I_{mh}\right\};\forall m,h\right\}}\left|\underset{m,h}{⋃}S_{mh}^{\left(i_{mh}\right)}\right|\text{.}$$

These statistical models are created to explain electricity demand variations in different seasons, helping identify essential variables. The selected variables for each seasonal situation are described in ${\hat{S}}_{mh}$.

### 3.3 Approach for estimating the hypothetical non-COVID-19 variable scenarios

**Fig. 9:**
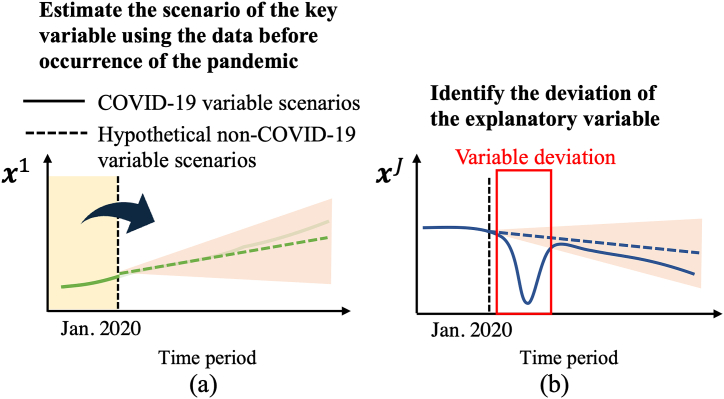
Estimation overview of the non-COVID-19 variable scenario for each explanatory variable: (a) Overview of the predicted variable and its confidence interval and (b) an example of the variable significantly diverged from their estimated scenarios during the pandemic.

For each variable that exhibited fluctuations during the pandemic, a non-COVID-19 scenario is estimated in Step 2, with deviations from this projected scenario subsequently analyzed. Fig. 9 provides an overview of how these variable scenarios are estimated. Specifically, using ARIMAX models, we forecast long-term variable scenarios using pre-pandemic data, which includes a predicted variable and its confidence interval, as shown in Fig. 9(a). We then focus on the discrepancies between these scenarios and the observed variables to determine which significantly diverged from their estimated scenarios during the pandemic, as illustrated in Fig. 9(b). The variables selected for scenario forecasting, clearly impacted by the pandemic, are monitored daily, monthly, or annually; Table A1 in the Appendix shows the observation granularity for each explanatory variable.

**Fig. 10:**
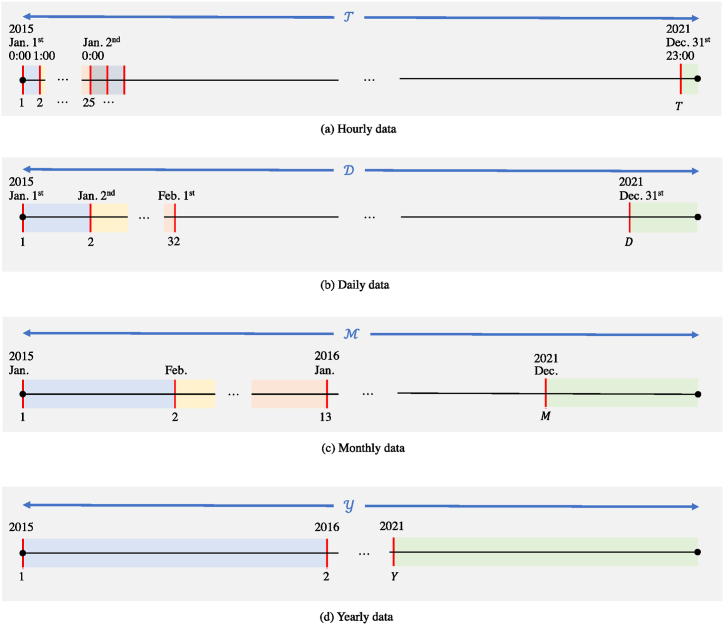
Overview of observation granularity for each variable: (a) Hourly data, (b) daily data, (c) monthly data and (d) yearly data.

Fig. 10(a)-(d) illustrate an overview of the observation granularity for each variable.

Let T=(1,…,T) be the training period, and T be the number of samples of hourly data. The training period for daily data, *such as* stock price*s*, is defined as D={1,…,D}, where D denotes the number of samples. Similarly, the training periods for monthly data, such as the index of production in services, are defined as M={1,…,M}, and the training periods for yearly data, such as power generation amounts, are defined as Y={1,…,Y}.

Focusing on the daily variable $x_{d}^{j}\;\left(d\in D\right)$, the ARIMAX (P,O,Q) model is utilized to describe the target variable using historical data and exogenous variables that reflect the month and day of the week. This is defined in Eqs. (7), (8), (9) as follows:

(7) $${x^{*}}_{d}^{j}=\sum_{p=1}^{P}\kappa_{p}{x^{*}}_{d-p}^{j}+\sum_{q=1}^{Q}\mu_{q}\varepsilon_{d-q}+\sum_{a\in A}\nu_{a}h_{a}\left(d\right)+\sum_{b\in B}\xi_{b}h_{b}\left(d\right)+\varepsilon_{d}\text{,}$$

(8) $${{x^{*}}_{d}^{j}=\Delta^{O}x}_{d}^{j}\text{,}$$

(9) $$\varepsilon_{d}∼N\left(0,\sigma_{d}^{2}\right)\text{,}$$

where $h_{a}\left(d\right)$ and $h_{b}\left(d\right)$ represent functions that generate dummy variables for the month A=(January,…,December) and the day of the week B=(Monday,…,Sunday), respectively, within the target period as Eq. (10), (11):

(10) $$h_{a}\left(d\right)=\left\{ \begin{array}{c} 1\;\text{if}\;\text{the}\;\text{target}\;\text{period}\;d\;\text{is}\;\text{in}\;\text{the}\;\text{target}\;\text{month}\;a\in A\\ 0\;\text{if}\;\text{the}\;\text{target}\;\text{period}\;d\;\text{is}\;\text{not}\;\text{in}\;\text{the}\;\text{target}\;\text{month}\;a\in A, \end{array}\right.$$

(11) $$h_{b}\left(d\right)=\left\{ \begin{array}{c} 1\;\text{if}\;\text{the}\;\text{target}\;\text{period}\;d\;\text{is}\;\text{in}\;\text{the}\;\text{target}\;\text{day}\;\text{of}\;\text{the}\;\text{week}\;b\in B\\ 0\;\text{if}\;\text{the}\;\text{target}\;\text{period}\;d\;\text{is}\;\text{not}\;\text{in}\;\text{the}\;\text{target}\;\text{day}\;\text{of}\;\text{the}\;\text{week}\;b\in B\;. \end{array}\right.$$

Additionally, $\Delta^{O}x_{d}^{j}$ denotes the O-order differentiated time series, $\sigma_{d}^{2}$ represents the variance of the white noise $\varepsilon_{d}$, and ${\kappa =(\kappa_{1},\ldots ,\kappa }_{P})$, ${\mu =(\mu_{1},\ldots ,\mu }_{Q})$, ${\nu =(\nu_{\text{January}},\ldots ,\nu }_{\text{December}})$, and ${\xi =(\xi_{\text{Monday}},\ldots ,\xi }_{\text{Sunday}})$ represent the sets of model coefficient parameters, κ denotes the set of coefficients for the autoregressive component, μ denotes the coefficients for the moving average component, and ν and ξ represent the coefficients for the exogenous variables corresponding to months and days of the week, respectively.

These parameters κ, μ, ν, and ξ are estimated based on maximum likelihood estimation (MLE) in Eq. (12), which is conceptually similar to the least-squares estimates as shown below [35]:

(12) $$\hat{\kappa },\hat{\mu },\hat{\nu },\hat{\xi }=\begin{array}{c} \mathrm{argmin}\\ \kappa ,\mu ,\nu ,\xi \end{array}\sum_{d\in D}{\left({x^{*}}_{d}^{j}-\sum_{p=1}^{P}\kappa_{p}{x^{*}}_{d-p}^{j}+\sum_{q=1}^{Q}\mu_{q}\varepsilon_{d-q}+\sum_{a\in A}\nu_{a}h_{a}\left(d\right)+\sum_{b\in B}\xi_{b}h_{b}\left(d\right)\right)}^{2},$$

where $\sigma_{d}$ is derived from the standard deviation of the residuals, which are the differences between the observed values and the fitted values from Eq. (7) using the estimated parameters, expressed in Eq. (13) as follows:

(13) $$\sigma_{d}=\text{sd}\left({x^{*}}_{d}^{j}-\sum_{p=1}^{P}\kappa_{p}{x^{*}}_{d-p}^{j}+\sum_{q=1}^{Q}\mu_{q}\varepsilon_{d-q}+\sum_{a\in A}\nu_{a}h_{a}\left(d\right)+\sum_{b\in B}\xi_{b}h_{b}\left(d\right)\right)\text{.}$$

The predicted value of ${\hat{x}}_{d}^{j}\;\left(d>D\right)$ can be estimated as a non-COVID-19 variable scenario during the pandemic, and the 95 % confidence interval is determined by the difference between ${\hat{x}}_{d,\text{upper}}^{j}$ and ${\hat{x}}_{d,\text{lower}}^{j}$ [36]. The confidence intervals are derived in Eq. (14), (15), (16) as follows:

(14) $${\hat{x}}_{d,\text{lower}}^{j}\leq {\hat{x}}_{d}^{j}\leq {\hat{x}}_{d,\text{upper}\;}^{j}\text{,}$$

(15) $${\hat{x}}_{d,\text{lower}}^{j}={\hat{x}}_{d}^{j}-1.96\sigma_{d}\text{,}$$

(16) $${\hat{x}}_{d,\text{upper}}^{j}={\hat{x}}_{d}^{j}+1.96\sigma_{d}\;\text{.}$$

The deviating variables are identified by comparing the actual variable $x_{d}^{j}$ with the bounds of the confidence interval as follows:

• ${\hat{x}}_{d,\text{lower}}^{j}\leq x_{d}^{j}\leq {\hat{x}}_{d,\text{upper}}^{j}$: variable j varies within the projected range of non-COVID variable scenarios.
• ${\hat{x}}_{d,\text{lower}}^{j}>x_{d}^{j}\cup {\hat{x}}_{d,\text{upper}}^{j}<x_{d}^{j}$: Variable j deviates from the expected scenario during the pandemic.

Similarly, the ARIMAX model (P,O,Q) for describing the monthly variable $x_{m}^{j}\;\left(m\in M\right)$ is defined in Eq. (17), (18) as:

(17) $${x^{*}}_{m}^{j}=\sum_{p=1}^{P}\kappa_{p}{x^{*}}_{m-p}^{j}+\sum_{q=1}^{Q}\mu_{q}\varepsilon_{m-q}+\sum_{a\in A}\nu_{a}h_{a}\left(m\right)+\varepsilon_{m}\text{,}$$

(18) $$\hat{\kappa },\hat{\mu },\hat{\nu },\hat{\xi }=\begin{array}{c} \mathrm{argmin}\\ \kappa ,\mu ,\nu ,\xi \end{array}\sum_{m\in M}{\left({x^{*}}_{m}^{j}-\sum_{p=1}^{P}\kappa_{p}{x^{*}}_{m-p}^{j}+\sum_{q=1}^{Q}\mu_{q}\varepsilon_{m-q}+\sum_{a\in A}\nu_{a}h_{a}\left(m\right)\right)}^{2}\text{.}$$

The scenarios during the pandemic are estimated based on ${\hat{x}}_{m}^{j}$ (m>M), derived in Eq. (19), (20), (21) as follows:

(19) $${\hat{x}}_{m,\text{lower}}^{j}\leq {\hat{x}}_{m}^{j}\leq {\hat{x}}_{m,\text{upper}}^{j}\text{,}$$

(20) $${\hat{x}}_{m,\text{lower}}^{j}={\hat{x}}_{m}^{j}-1.96\sigma_{m}\text{,}$$

(21) $${\hat{x}}_{m,\text{upper}}^{j}={\hat{x}}_{m}^{j}+1.96\sigma_{m}\text{.}$$

The ARIMAX (P,O,Q) model for describing the yearly variable $x_{y}^{j}\;\left(y\in Y\right)$ is defined in Eq. (22), (23) as:

(22) $${x^{*}}_{y}^{j}=\sum_{p=1}^{P}\kappa_{p}{x^{*}}_{y-p}^{j}+\sum_{q=1}^{Q}\mu_{q}\varepsilon_{y-q}+\varepsilon_{y}\text{,}$$

(23) $$\hat{\kappa },\hat{\mu },\hat{\nu },\hat{\xi }=\begin{array}{c} \mathrm{argmin}\\ \kappa ,\mu ,\nu ,\xi \end{array}\sum_{y\in Y}{\left({x^{*}}_{y}^{j}-\sum_{p=1}^{P}\kappa_{p}{x^{*}}_{y-p}^{j}+\sum_{q=1}^{Q}\mu_{q}\varepsilon_{y-q}\right)}^{2}\text{.}$$

The scenarios during the pandemic are further estimated based on ${\hat{x}}_{y}^{j}$ (y>Y), expressed in Eq. (24), (25), (26) as follows:

(24) $${\hat{x}}_{y,\text{lower}}^{j}\leq {\hat{x}}_{y}^{j}\leq {\hat{x}}_{y,\text{upper}}^{j}\text{,}$$

(25) $${\hat{x}}_{y,\text{lower}}^{j}={\hat{x}}_{y}^{j}-1.96\sigma_{y}\text{,}$$

(26) $${\hat{x}}_{y,\text{upper}}^{j}={\hat{x}}_{y}^{j}+1.96\sigma_{y}\text{.}$$

### 3.4 Evaluation of additive contribution to demand gap

**Fig. 11:**
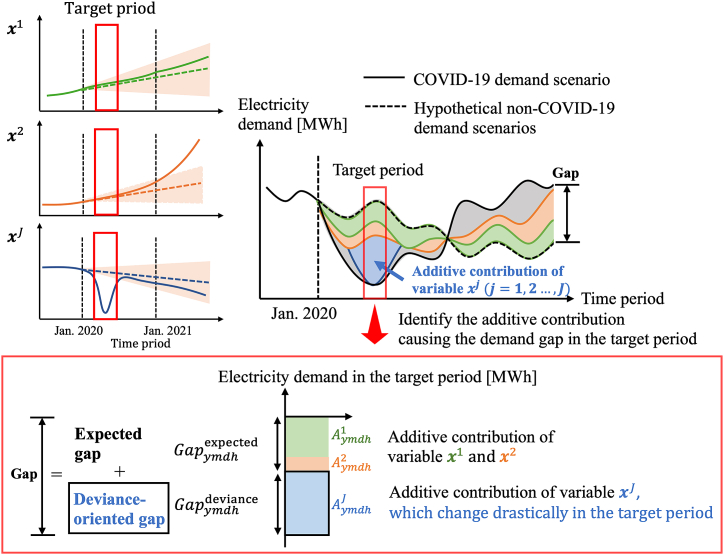
Analysis of the impacts of key variables on the electricity demand gap.

In Step 3, we analyze the demand gap between COVID-19 and non-COVID-19 demand scenarios using PLAMs constructed in Step 2. Fig. 11 shows an overview of this analysis. The demand gap can be classified into two components: *the deviance-oriented gap*, caused by significant changes in variables from their non-COVID-19 behavior, and *the expected gap*, resulting from variables acting according to typical non-COVID-19 scenarios. In Fig. 11, variable $x^{J}$ deviates from its non-COVID-19 variable scenario in the target period, leading to a deviance-oriented gap.

Here, we calculate the additive contributions of each variable over different seasonal periods, considering their significant fluctuations throughout the target period. Let $Gap_{ymdh}$ be the electricity demand gap at hour h on day d in month m of year y. The non-COVID-19 demand scenario, the COVID-19 demand scenario, and the demand gap between these are outlined using the constructed model in Step 1 (Eq. (1)) as detailed in Eq. (27), (28), (29):

(27) $${\hat{l}}_{ymdh}^{\text{nonCOVID}}=f\left({\hat{x}}_{ymdh};\theta \right),$$

(28) $${\hat{l}}_{ymdh}^{\text{COVID}}=f\left(x_{ymdh};\theta \right)\text{,}$$

$$Gap_{ymdh}={\hat{l}}_{ymdh}^{\text{COVID}}-{\hat{l}}_{ymdh}^{\text{nonCOVID}}$$

(29) $$=\sum_{j\in {\hat{S}}_{mh}}\left(\left(\beta_{j}+\tau_{j,1}\;\right)\left({x^{*}}_{ymdh}^{j}-{\hat{x}}_{ymdh}^{j}\right)+\sum_{k=2}^{K}\tau_{j,k}\left({\varphi_{j}^{k}(x^{*}}_{ymdh}^{j})-\varphi_{j}^{k}\left({\hat{x}}_{ymdh}^{j}\right)\right)\right))\text{,}$$

where ${\hat{S}}_{mh}\subseteq S$ represents the index subset selected by the enumerated sparse PLAM for each seasonal situation in Step 1. Thus, the additive contribution of variable j can be described in Eq. (30) as follows:

(30) $$A_{ymdh}^{j}=\left(\beta_{j}+\tau_{j,1}\;\right)\left({x^{*}}_{ymdh}^{j}-{\hat{x}}_{ymdh}^{j}\right)+\sum_{k=2}^{K}\tau_{j,k}\left({\varphi_{j}^{k}(x^{*}}_{ymdh}^{j})-\varphi_{j}^{k}\left({\hat{x}}_{ymdh}^{j}\right)\right))\text{.}$$

The pandemic-induced demand gap is categorized into two types: the deviance-oriented gap, where variables behave abnormally compared to non-COVID-19 conditions, and the expected gap, where variables fluctuate within expected ranges. These gaps are quantified based on the additive contributions of variables, detailed in Eq. (31), (32), (33), (34), (35) as follows:

(31) $$Gap_{ymdh}=Gap_{ymdh}^{\text{expected}}+Gap_{ymdh}^{\text{deviance}}\text{,}$$

(32) $$Gap_{ymdh}^{\text{deviance}}=\sum_{j\in {\hat{S}}_{\;ymdh}^{\text{deviance}}}A_{ymdh}^{j}\text{,}$$

(33) $$Gap_{ymdh}^{\text{expected}}=\sum_{j\in {\hat{S}}_{ymdh}^{\text{expected}}}A_{ymdh}^{j}\text{,}$$

(34) $${\hat{S}}_{\;mh}^{\text{deviance}}\cup {\hat{S}}_{\;mh}^{\text{expected}}={\hat{S}}_{mh}\text{,}$$

(35) $${\hat{S}}_{\;mh}^{\text{deviance}}\cap {\hat{S}}_{\;mh}^{\text{expected}}=\emptyset \text{,}$$

where ${\hat{S}}_{\;mh}^{\text{deviance}}$ represents the indices of key variables that deviate from their respective scenarios during each seasonal period, and ${\hat{S}}_{\;mh}^{\text{expected}}$ denotes the variables that vary within the expected scenario range. These variables are identified by analyzing the differences between the non-COVID-19 variable scenario and the actual variable values, as outlined in Step 2.

## 4 Case study

### 4.1 Simulation setup

The analysis of the pandemic's demand gap in Germany utilizes actual hourly electricity usage data from January 2015 to December 2021. This analysis includes 179 explanatory variables related to categories such as weather, interest rates, stock prices, calendars, and GDP data, as detailed in Table 2. For constructing the sparse PLAMs, we have to tune parameter K in Eq. (2) for nonlinear transformation and parameter λ in Eq. (4) for the sparse scheme. The parameters were determined through a one-day walk-forward validation [33] spanning from January 2018 to December 2021. During this period, statistical models were reconstructed daily and evaluated based on the forecasts of the subsequent day. In the enumeration scheme of Step 1, we utilized the parameter ε=0.005, which controlled the number of enumerated models. This rigorous approach ensures precise identification and analysis of the variables impacting the electricity demand gap during the pandemic.

We examined four distinct models outlined in Table 3 to analyze electricity demand. The Linear Model (LM) uses a limited set of explanatory variables—such as weather conditions, the German stock index, and holiday/weekday data—which are commonly employed in traditional studies [6,7], and assumes a linear relationship among these variables. Similarly, Additive Model 1 (AM1) also utilizes a limited number of variables but introduces considerations for nonlinearity among them. Conversely, Additive Model 2 (AM2) incorporates a broader array of variables (179 variables) and is used in more recent studies [5,10] to model nonlinearity among these variables. Furthermore, the PLAM engages many variables to implement the procedure outlined in Section 3 and identifies a small set of annually dominant variables. The evaluation of these models was conducted based on their descriptive accuracy.

**Table 3:** Conditions of the constructed models.

Item	Variables	Note
LM	Weather, German stock index, holiday/weekday dummy (Limited number of explanatory variables)	Utilize linear models, considering linear relationships among variables
AM1		Utilize additive models, considering nonlinear relationships among variables
AM2	All explanatory variables in Table 2	
PLAM		Utilize PLAMs, assuming linear/nonlinear relationships among variables

### 4.2 Description accuracy of constructed models

**Fig. 12:**
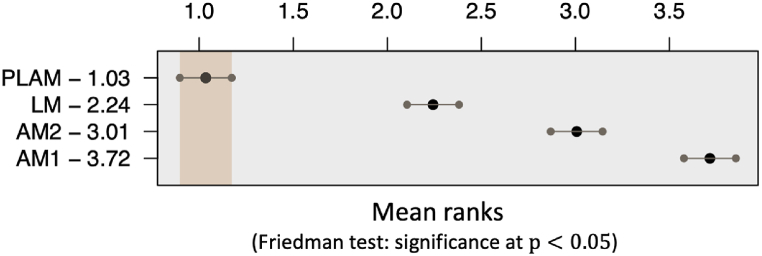
Results of the Nemenyi test assessing the average ranks of description accuracy. Hatching intervals indicate no significant differences between average ranks were found based on the test results.

Initially, we developed statistical models of hourly electricity demand using both naive and advanced approaches listed in Table 3, based on the dataset. The performance of the constructed PLAMs in capturing the behavior of electricity demand is substantiated by the average root-mean-squared error (RMSE) and Nemenyi tests. Table 4 presents the RMSE values used to compare the descriptive accuracy during the training period. The RMSE is calculated, defined in Eq. (36) as follows:

(36) $$RMSE=\sqrt{\frac{1}{T}\sum_{ymdh}{\left(l_{ymdh}-f\left(x_{ymdh};\theta \right)\right)}^{2}}$$

where T denotes the number of data samples. Fig. 12 presents the outcomes of the post-hoc Nemenyi test [43], commonly employed to evaluate significant differences in descriptive ranks based on their RMSE values. This analysis assigns the model with the lowest RMSE the highest rank. The Nemenyi test compares the average ranks across multiple models and identifies significant performance disparities among them. The results reveal that models AM1 and AM2 exhibited lower descriptive accuracy than model LM. This implies that enhancing the assumptions regarding linear or nonlinear relationships between the demand and the variables, and careful selection of explanatory variables could improve descriptive accuracy. Notably, model PLAM demonstrated higher descriptive accuracy than conventional naive modeling approaches, indicating that the proposed method effectively identifies key variables and discerns the relationships of linearity or nonlinearity between them.

**Table 4:** RMSE of the models as described in Table 2.

Model	RMSE [MWh]
LM	9133.1
AM1	22777.0
AM2	11073.7
PLAM	6804.2

### 4.3 Analysis of the gap caused by the pandemic

**Fig. 13:**
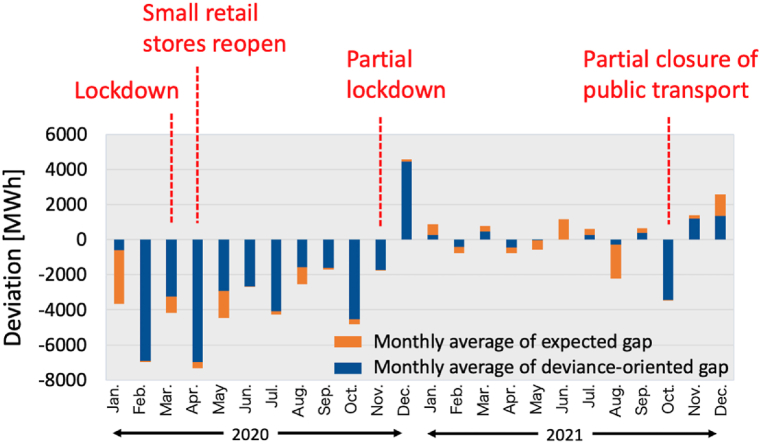
Monthly averages of the expected gap and deviance-oriented gap.

**Fig. 14:**
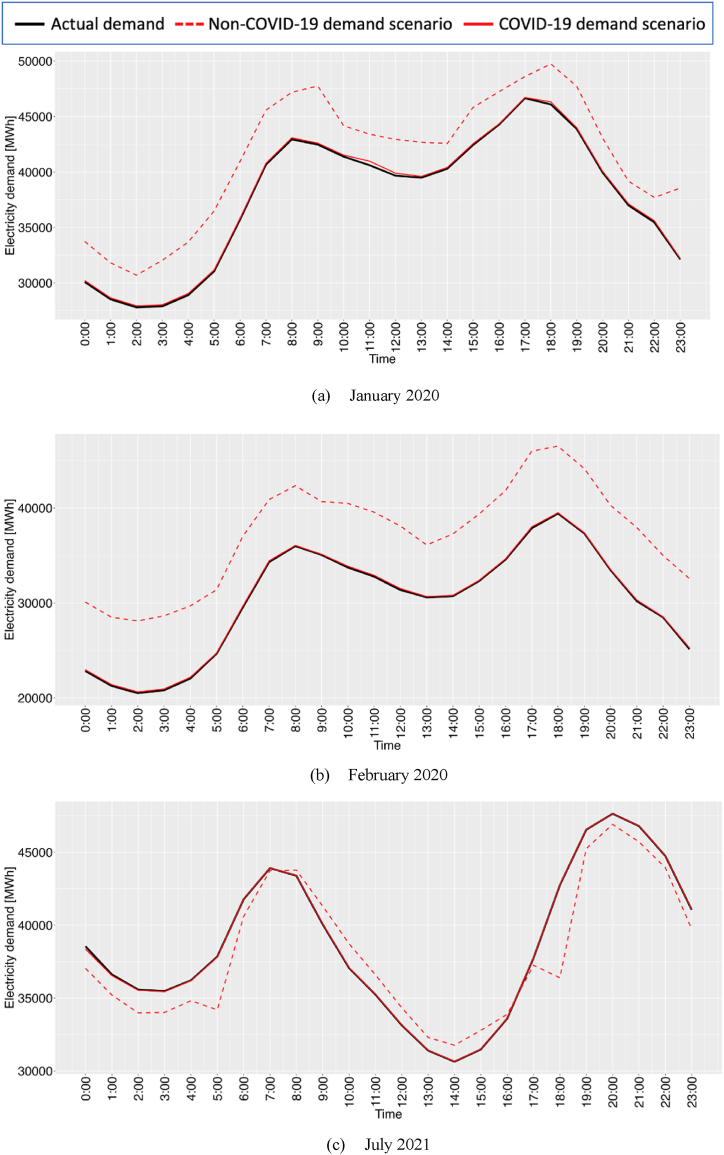
Hourly averages of actual, COVID-19, and non-COVID-19 demand scenarios (a) in January 2020, (b) in February 2020 and (c) in July 2021.

The analysis also examines the impact of the pandemic on seasonal variations. Fig. 14 displays the monthly averages of the expected and deviance-oriented gaps. In response to the COVID-19 outbreak, Germany implemented its initial nationwide lockdown in March 2020. A phased mitigation strategy followed, with the government announcing on April 15 that small retail stores would reopen on April 20, and schools would gradually begin to reopen from May 4. However, due to a resurgence in case numbers, a partial lockdown was reinstated from November 2, 2020, to March 1, 2021 [44]. Fig. 13 indicates that the deviance-oriented gap varies seasonally, showing that in April 2020, when the most substantial gap occurred, approximately 95 % of this gap resulted from the deviance-oriented demand gap, aligning with the initial lockdown measures. Further analysis suggests that despite the continuation of lockdown measures, Germany experienced a notable recovery in electricity demand from late 2020 into 2021. These findings indicate that the proposed approach effectively captures behavioral changes in the demand gap unaccounted for by movement restriction policies alone.

This section presents an analysis of hourly averages for the actual COVID-19 demand scenario alongside a hypothetical non-COVID-19 scenario, as depicted in Fig. 14(a)–(c). The results demonstrate that the proposed approach successfully reproduced the COVID-19 demand scenario through the construction of probabilistic latent additive models (PLAMs). Additionally, the PLAMs generated hourly hypothetical non-COVID-19 scenarios, revealing fluctuations in the hourly demand gap dependent on the time of day. In 2020, a consistent trend of decreasing electricity demand was observed across all time slots. Conversely, the 2021 data show a recovery in electricity demand, marked by increases during early morning and late-night hours and decreases during daylight hours. This pattern may indicate a shift in daily routines across commercial, industrial, and residential sectors and adaptation to remote working practices, which consequently alter traditional electricity consumption peaks. These observations suggest that the nature of electricity usage varies with the pandemic conditions.

### 4.4 Discussion of selected variables

**Fig. 15:**
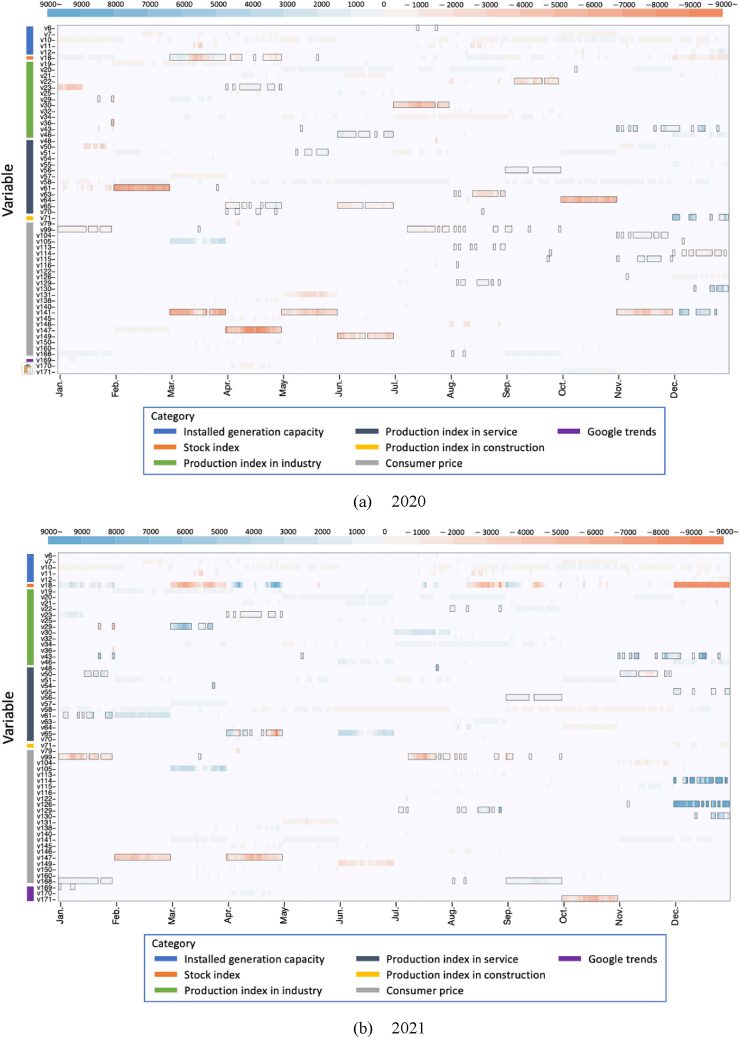
Mean additive contributions of each key variable affecting the electricity demand gap (a) in 2020 and (b) in 2021. The black box highlights variables that deviate from the non-COVID-19 scenario during the target period. The x-axis represents the transition of the situation (m,h), and the y-axis represents the selected variables. The color bar on the y-axis indicates the categories of the variable. (For interpretation of the references to color in this figure legend, the reader is referred to the Web version of this article.)

Furthermore, we examine the essential variables influencing the demand gap during the pandemic and assess the additive contributions of each variable. The situation-dependent modeling approach selected 72 out of 179 explanatory variables to characterize the annual demand behavior for each monthly hour. We highlight the variables significantly impacting the expected and deviance-oriented gaps, excluding weather and day-of-week factors that remained constant throughout the pandemic. Table 5 (a) displays the index of the variable ${\hat{S}}_{\;mh}^{\mathrm{deviance}}$ deviating from the non-COVID-19 scenario. Table 5 (b) lists the index and names of the key variable ${\hat{S}}_{\;mh}^{\mathrm{expected}}$ as they vary according to the non-COVID-19 scenario during the target period. Additionally, we explore the differences in the additive contributions of these key variables to the electricity demand gap across each month and hour. Fig. 15(a) and (b) illustrate the mean additive contributions for each seasonal period of 2020 and 2021; the contributions are derived in Eq. (37) as follows:

(37) $${\bar{A}}_{ymh}=\sum_{d}A_{ymdh}^{j}\text{,}$$

**Table 5a:** Key variables that deviated drastically from the projected scenarios.

Category	Index	Item
Installed generation capacity	6	“Other renewables^a^"
Stock index	18	“German Stock Index"
Production in industry	20	“Extraction of crude petroleum and natural gas"
	22	“Mining support service activities"
	23	“Manufacture of food products"
	29	“Manufacture of wood and products of wood and cork, except furniture; manufacture of articles of straw and plaiting materials"
	30	“Manufacture of paper and paper products"
	36	“Manufacture of other non-metallic mineral products^b^"
	43	“Manufacture of other transport equipment^c^"
	46	“Repair and installation of machinery and equipment"
Production in service	48	“Wholesale and retail trade and repair of motor vehicles and motorcycles"
	50	“Land transport and transport via pipelines"
	51	“Water transport"
	54	“Postal and courier activities"
	55	“Publishing activities"
	56	“Motion picture, video and television programme production, sound recording and music publishing activities"
	61	“Legal and accounting activities"
	63	“Advertising and market research"
	64	“Other professional, scientific and technical activities^d^"
	65	“Rental and leasing activities"
	70	“Office administrative, office support and other business support activities"
Production in construction	71	“Buildings"
Consumer price	99	“Refuse collection"
	104	“Liquid fuels"
	105	“Solid fuels"
	113	“Major tools and equipment"
	114	“Small tools and miscellaneous accessories"
	115	“Nondurable household goods"
	116	“Domestic services and household services"
	126	“Spare parts and accessories for personal transport equipment"
	129	“Other services in respect of personal transport equipment^e^"
	130	“Passenger transport by railway"
	141	“Recording media"
	147	“Equipment for sport, camping and open-air recreation"
	149	“Pets and related products"
Google Trends	168	“energy efficiency”
	169	“global warming”
	171	“solar energy”

a This category includes the amount of power generated by renewable energy resources but does not include the amount generated from biomass, hydropower, wind offshore, wind onshore, and photovoltaics.

b This category includes manufacturing activities related to a single substance of mineral origin, e.g., the manufacturing of glass and glass products (e.g., flat glass, hollow glass, fibers, and technical glassware), ceramic products, tiles and baked clay products, and cement and plaster from raw materials to finished articles.

c This category includes manufacturing of transportation equipment such as ship-building and boat manufacturing, manufacturing of railroad-rolling stock, locomotives, air, spacecraft, and related components.

d This category includes diverse service activities generally delivered to commercial clients. This includes activities for which more advanced professional, scientific, and technical skill levels are needed, but it does not include ongoing, routine business functions typically of short duration.

e This category includes hiring garages or parking spaces that do not provide parking related to dwellings.

**Table 5b:** Key variables fluctuating with scenarios.

Category	Index	Item
Installed generation capacity	7	"Nuclear"
	10	"Fossil gas"
	11	"Hydropumped storage"
	12	"Other conventional generation^a^"
Production in industry	19	"Mining of coal and lignite"
	21	"Other mining and quarrying^b^"
	25	"Manufacture of tobacco products"
	32	"Manufacture of coke and refined petroleum products"
	34	"Manufacture of basic pharmaceutical products and pharmaceutical preparations"
Production in service	57	"Programming and broadcasting activities"
	58	"Telecommunications"
Consumer price	79	"Fruit"
	122	"Paramedical services"
	131	"Passenger transport by road"
	138	"Equipment for reception, recording, and reproduction of sound and picture"
	140	"Information processing equipment"
	145	"Maintenance and repair of other major durables for recreation and culture"
	146	"Games, toys, and hobbies"
	150	"Veterinary and other services for pets"
	160	"Electrical appliances for personal care"
Google trend	170	"Renewable energy"

a This category includes the amount of conventional power generation but not the generation from nuclear, lignite, hard coal, fossil gas, and hydro-pumped storage.

b This category includes the mining and quarrying various minerals and materials: abrasive materials, asbestos, siliceous fossil meals, natural graphite, steatite (talc), and feldspar.

The black box highlights the variable ${\hat{S}}_{\;mh}^{\mathrm{deviance}}$ deviating from the non-COVID-19 scenario during the target period.

These findings elucidate the variations in additive contributions across different seasonal conditions, defined by the sets of year y, month m, and hour h. The analysis identified several variables as pivotal despite their stability during the pandemic. Notably, the installed capacity of power generation (variables v7, v10, v11, v12), the manufacturing of basic pharmaceutical products and pharmaceutical preparations (v34), and the consumer price of passenger transport by road (v131) were deemed essential. Furthermore, certain variables significantly influenced the deviance-oriented gap, including the production of land transport and transport via pipelines (v50), postal and courier activities (v54), consumer prices of domestic and household services (v116), passenger transport via railways (v130), and equipment for sport, camping, and open-air recreation (v147). The general trend suggests that these variables are susceptible to fluctuations driven by lockdown measures and increased home-based activities during the pandemic, potentially leading to substantial demand gaps. These variations may also differ across seasons. For example, in March 2020, the consumer price of recording media (v141) diverged from the non-COVID-19 scenario, significantly influencing the demand gap. However, by 2021, the impact of these variables on the demand gap had diminished, suggesting a shift towards an expected scenario-dependent explanation of the gaps observed.

**Fig. 16:**
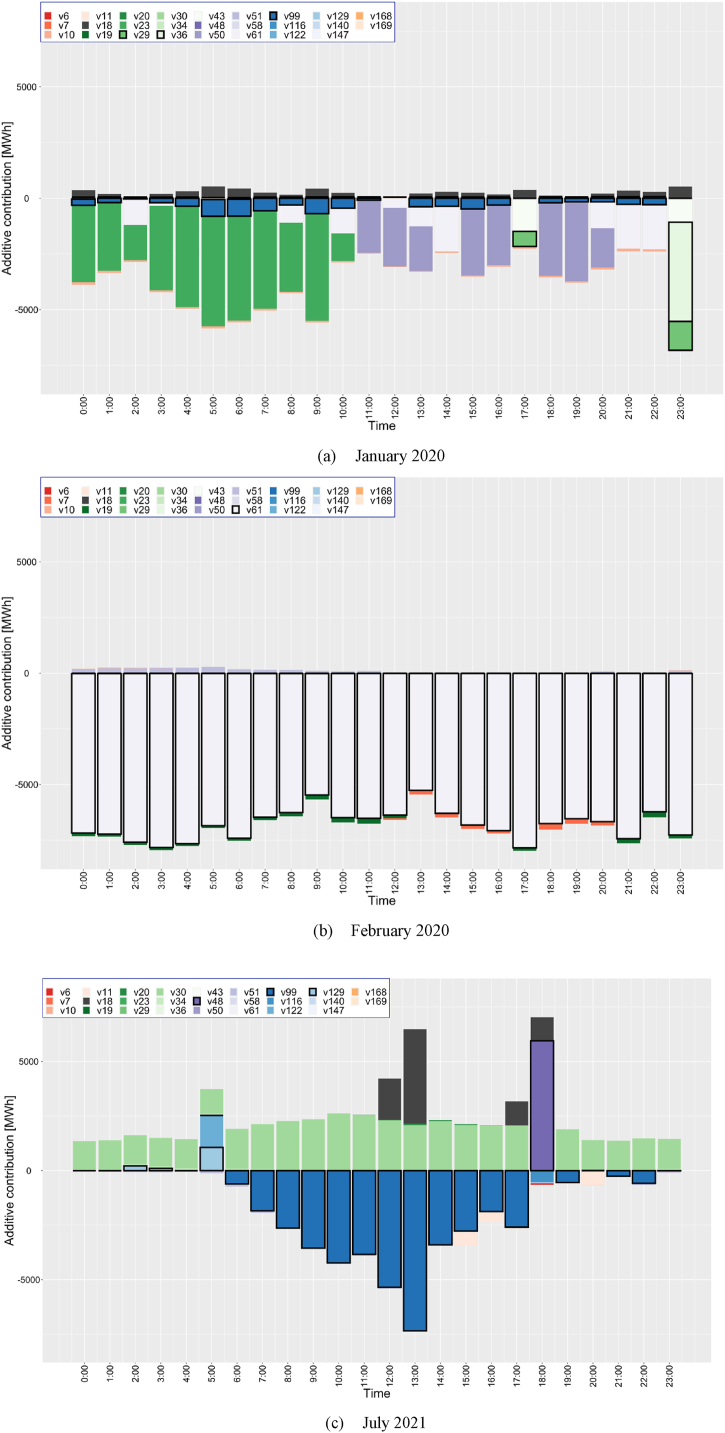
Examples of additive contributions of crucial variables for the period shown in Fig. 14: (a) January 2020, (b) February 2020 and (c) July 2021. The black box illustrates key variables and their contributions to the demand gap relative to the deviance-oriented gap.

Fig. 16 shows examples of the additive contributions of key variables for the same period covered in Fig. 15. The additive contributions are calculated based on the mean values of $A_{ymdh}^{j}$ from Eq. (30) for each year, month, and hour to explain the demand gap. As illustrated in Fig. 16 (a), the manufacturing of food products (v23) and the production of land transport and transport via pipelines (v50) in January 2020 had a significant impact on the demand gap. These variables did not exhibit considerable deviations during the target period, suggesting that the demand gap in January 2020 was likely unrelated to the pandemic. Notably, COVID-19 infections in Germany commenced towards the end of January, indicating that the pandemic had minimal influence on demand during this month.

In contrast, as depicted in Fig. 16 (b), the influence of variables diverging from the non-COVID-19 variable scenario during the pandemic substantially increased in February 2020 compared to January. For example, the production of legal and accounting activities (v61) had a pronounced effect on the demand gap as this sector was severely impacted by the pandemic, leading to a downturn in business activities due to restrictions [45]. In July 2021, as portrayed in Fig. 16(c), the manufacturing of paper and paper products (v30) continued to be crucial. Additionally, the consumer price of refuse collection (v99) experienced significant deviations during daytime hours (6:00 to 17:00), highlighting the substantial impact of variable deviations during these hours in the target month. This finding suggests that the additive contributions of key variables due to their deviations significantly altered their behavior according to the season and time of day. These seasonal and temporal variations observations illustrate idiosyncratic changes significantly driven by pandemic-induced economic conditions and consumer behaviors. To accurately predict the long-term behavior of electricity demand, it is imperative to thoroughly characterize the key variables and their behaviors under specific seasonal circumstances. To ensure reliable forecasts, these variables must be accurately modeled considering prevailing economic conditions and consumer interests.

**Fig. 17:**
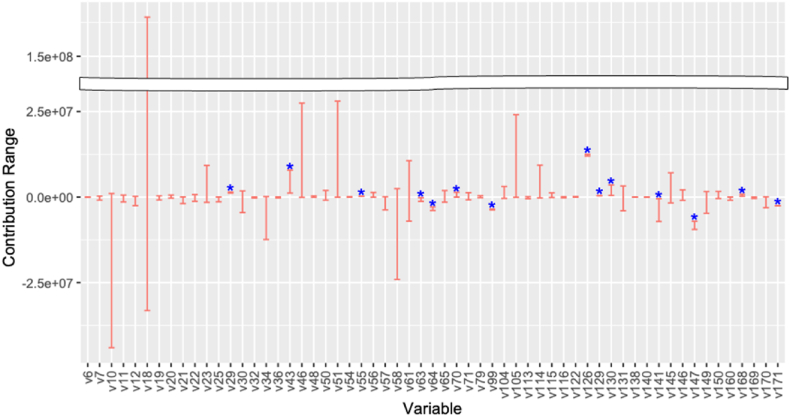
Range of additive contributions of each key factor of non-COVID-19 scenario variation. Blue asterisks indicate that the variables in the upper and lower bounds have the same sign. (For interpretation of the references to color in this figure legend, the reader is referred to the Web version of this article.)

**Fig. A1:**
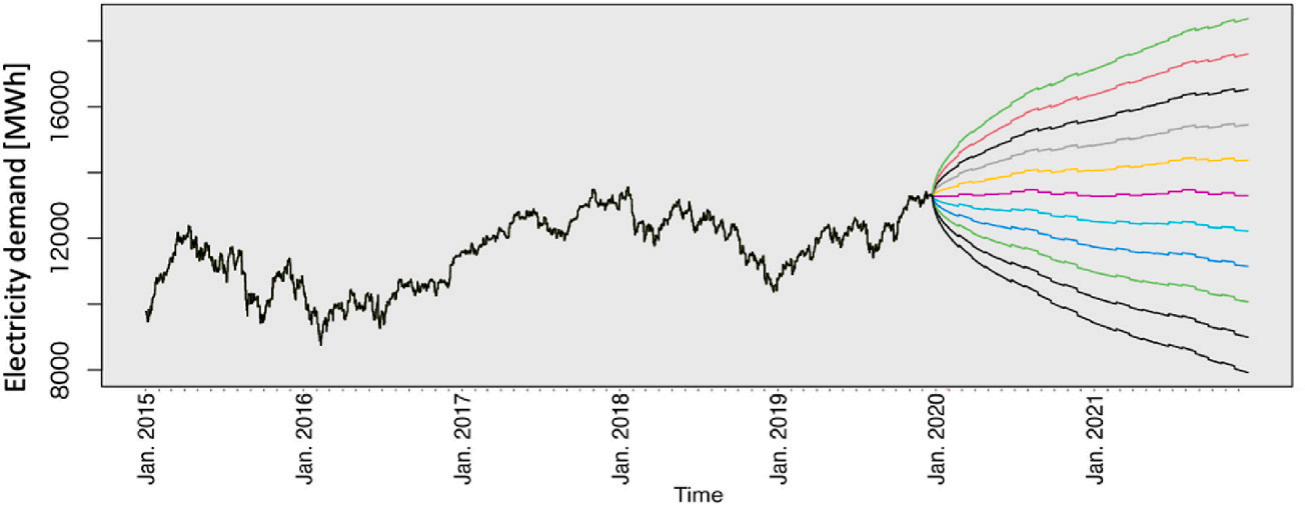
An example of the variation scenarios of German stock index (DAX).

Furthermore, we explore the confidence interval analysis introduced for each key variable in Section 3.3, focusing on the fluctuations in additive contributions attributed to uncertainty in the explanatory variables. We specifically concentrate on the variation in each non-COVID-19 variable scenario and examine their implications on the variability of additive contributions impacting the demand gap. The variation scenarios are derived from 0 to 100 percent tiles within the confidence interval; an example of these scenarios for a specific variable is illustrated in Fig. A1 in the Appendix. For each variation scenario, we analyze the range of contributions to the variable variation using the same process for deriving the additive contribution presented in Section 3. Fig. 17 shows the range of additive contributions of each key factor. In this figure, variables marked with a blue asterisk indicate that the upper and lower bounds have the same sign. It suggests that these variables consistently influence the electricity demand gap under factor uncertainty. Such results provide relatively reliable information for electricity utilities and governments as a factor in capturing electricity demand trends. Conversely, other variables exhibit erratic behavior, with their impacts fluctuating positively or negatively in response to uncertainties. Although these variables are expected to contribute to electricity demand, the direction and magnitude of their contributions may vary significantly due to their factor variability. This requires careful consideration by utilities and governments when analyzing data and forecasting demand.

### 4.5 Analysis of the factors affecting the gap caused by the pandemic

We analyze the trends in the additive contributions of each critical variable that influenced the deviance-oriented gap ${A_{ymdh}^{j}\;(\forall j\in \hat{S}}_{\;mh}^{\mathrm{deviance}})$. We employ principal component analysis (PCA) [46] to extract the temporal trends of the significant patterns of contributions constituting the deviance-oriented gap. In this context, $pc\left(q\right)=\left\{{pc}_{ymdh}^{q}\right\}$ represents the q th principal component, and $\Psi_{q}^{j}$ is the factor loading of variable $x^{j}$ in the q th principal component. The ${pc}_{ymdh}^{q}$ values are computed, defined in Eq. (38) as follows:

(38) $${pc}_{ymdh}^{q}=\sum_{j}\Psi_{q}^{j}x_{ymdh}^{j}\text{.}$$

**Fig. 18:**
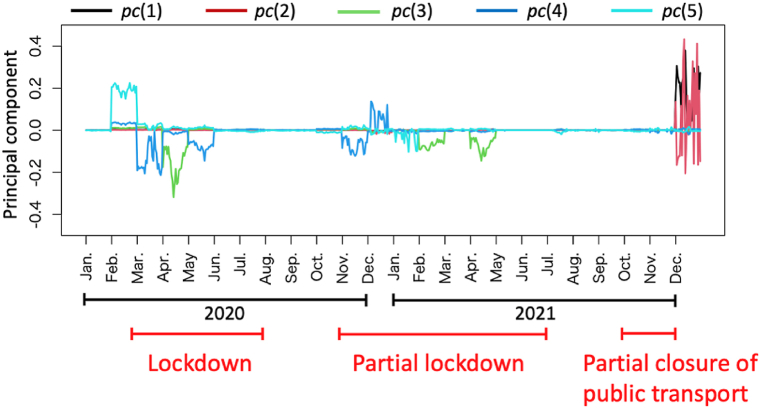
Five major patterns of contributions that constitute the deviance-oriented gap.

Fig. 18 shows the five principal patterns of contributions that characterize the deviance-oriented gap during the target period, illustrating distinct patterns influenced by government regulations and changes in consumer behavior due to the pandemic. For instance, the significant patterns pc(1) and pc(2) seem significantly correlated with the partial closure of public transport, while pc(2) also relates strongly to the general lockdown measures. Notably, pc(5) is prominent in the early stages of the pandemic.

**Fig. 19:**
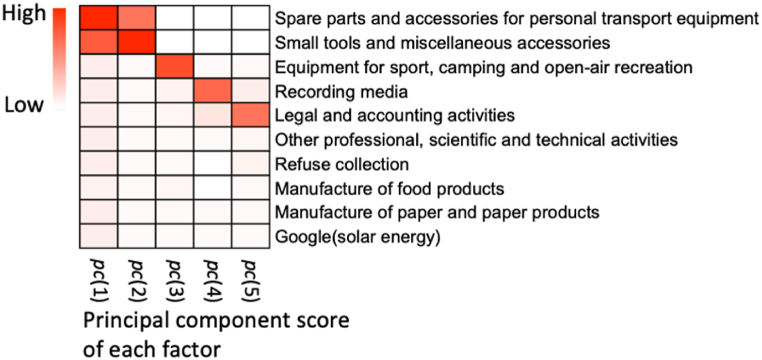
Principal component scores of critical factors to each significant pattern. The top 10 variables with the greatest influence on the pattern are shown. The intensity of the color of the squares indicates the magnitude of the impact of each variable category on the electricity demand gap. (For interpretation of the references to color in this figure legend, the reader is referred to the Web version of this article.)

Further analysis of the key factors influencing these patterns is presented in Fig. 19, which details the principal component scores of specific variables to each significant pattern; we focus on the top 10 variables with the most critical influence on the significant pattern. The results highlight the substantial impact of the consumer price of small tools and miscellaneous accessories (v114) and spare parts and accessories for personal transport equipment (v126) in patterns pc(1) and pc(2). This suggests that changes in demand for personal mobility resources significantly influenced the deviance-oriented gap during the partial closure of public transport. The prominence of the consumer price of equipment for sports, camping, and open-air recreation (v147) in pc(3) indicates that the demand for outdoor equipment predominantly affected the demand gap during lockdown periods. Additionally, the influence of the production of legal and accounting activities (v61) is a significant driver in pc(5), suggesting that the demand gap was responsive to variations in business and government operations during the pandemic. These findings demonstrate the efficacy of using PCA to discern diverse patterns in the deviance-oriented demand gap resulting from various pandemic-related events and to pinpoint the key factors associated with each pattern.

**Fig. 20:**
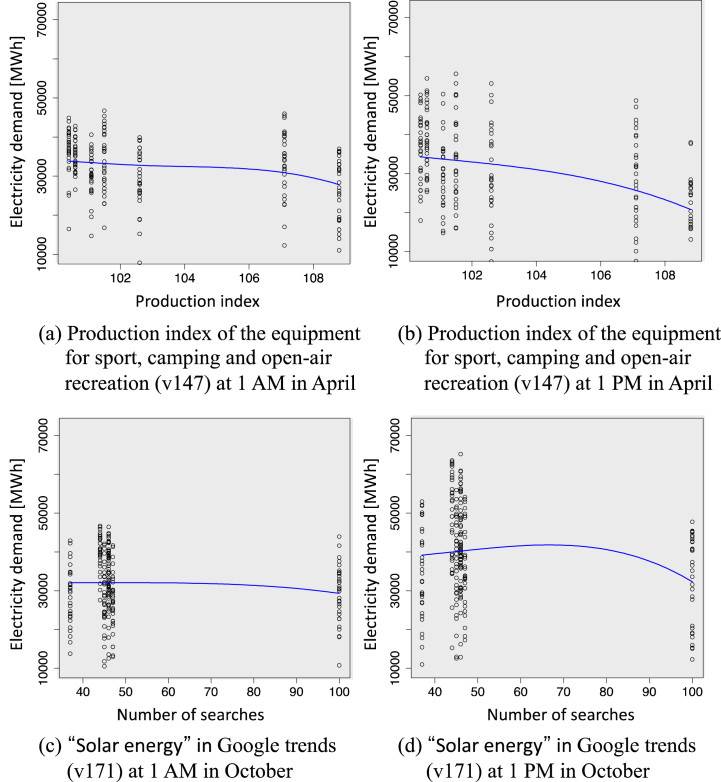
Relationships between seasonal electricity demand and key factors. Solid lines represent curves derived from the sparse PLAM introduced in Section 3: (a) Production index of the equipment for sport, camping and open-air recreation at 1 a.m. in April and (b) at 1 p.m. in April, and (c) “Solar energy” in Google trends at 1 a.m. in October and (d) at 1 p.m. in October.

Lastly, we focus on the production index of the equipment for sport, camping and open-air recreation (v147) and the term "solar power" used in Google trends (v171) in Fig. 19 and analyze relationships between the electricity demand and key factors; these variables are confirmed to have that a consistent trend of the impact on the demand gap under factor variation in Fig. 17. Fig. 20(a) and (b) show the monotonically decreasing trend in electricity demand with increasing the production index of the equipment for sport, camping and recreation. These results suggest that outdoor activities have changed the behavior of electricity demand downward. Fig. 20(c) and (d) show that the electricity demand tends to decrease depending on the increasing number of searches for "solar energy" in Google Trends; the trend becomes noticeable during the day. These results suggest that increased consumer interest in solar power may contribute to reduced electricity demand. A plausible interpretation for this occurrence could be the rise in small-scale solar power installations, which has occurred with heightened consumer engagement and curiosity. Alternatively, it may be that consumers' growing concern about energy issues has led them to reduce their electricity consumption. Throughout the COVID-19 pandemic, there has been a marked shift in consumer attention toward energy issues, with an increase in engagement with renewable energy and electric vehicles [47]. This shift in focus has indirectly led consumers to adopt behaviors that reduce electricity demand.

The results suggest that our proposed data-driven analysis method allows for the mechanical identification of key variables associated with the electricity demand gap from a wide range of possible variables. Furthermore, by focusing on a select few key factors, our analysis of the additive contribution of each variable to the gap allows for a qualitative understanding of the relationships between the factor and the demand gap, relationships that have not conventionally been considered.

## 5 Conclusions

In this study, we developed a methodology to ascertain the impact of key variables on the electricity demand gap in Germany during the COVID-19 pandemic. Utilizing ARIMAX models, we quantified deviations of each explanatory variable from hypothetical non-COVID-19 variable scenarios. Additionally, we implemented a sparse enumerated PLAM to construct demand models that facilitate the selection of key variables and elucidate demand behaviors. This dual approach, employing both ARIMAX and PLAMs, effectively pinpointed the variables influenced by shifts in economic conditions and consumer behaviors throughout the pandemic, and it delineated their impact on the electricity demand gap across different seasons. Key findings of this research include.

• The ARIMAX model proved instrumental in estimating non-COVI-19 variable scenarios absent of COVID-19 influences during the pandemic, providing a baseline for comparative analysis.
• Our variable selection methodology, which utilizes a sparse enumeration technique, adeptly identified critical variables that characterize electricity demand fluctuations across various seasonal contexts.
• Using PLAMs facilitated a detailed understanding of the interrelationships between electricity demand and pivotal variables under diverse seasonal conditions.
• Ultimately, our approach highlighted the primary effects of significant variables on the demand gap instigated by the COVID-19 pandemic, underscoring the responsiveness of the electricity sector to external disruptions.

This comprehensive analysis underscores the value of advanced modeling techniques in understanding complex market dynamics and aiding policymakers and industry leaders in making informed decisions during unprecedented times.

### 5.1 Policy implications

In this study, we focused on the electricity demand gap in Germany, analyzing the influence of the pandemic on changes in electricity demand. This analysis is instrumental in understanding the dynamics behind these changes and in forecasting future demand behaviors. Such insights are crucial for strategic decisions regarding infrastructure investment, such as enhancing grid flexibility to adapt to electricity use shifts and creating informed public messages about energy conservation and management during crisis events. For instance, electricity utilities and governments can use these insights to adjust energy supply strategies, ensuring stability in electricity provision despite unpredictable demand fluctuations. Moreover, this analysis supports developing and promoting energy efficiency initiatives tailored to the observed shifts in consumption patterns. The influence of various factors on the demand gap may fluctuate with changes in government policies and other significant social events. Therefore, it is essential for electricity utilities and governments in each country and region to conduct detailed analyses focusing on individual power demands and variables. Moreover, it is imperative to perform demand analysis and estimation to prepare for future crisis events that could impact demand. This study proposes a general modeling approach that facilitates the analysis of demand factors based on a consistent methodology applicable to any period and observed variables. Electricity utilities and governments should continue to compare data from multiple perspectives, including different geographic regions and periods, especially during crisis events. This comprehensive approach will enhance the resilience and responsiveness of energy systems to global challenges.

### 5.2 Study limitations and future recommendations

In this study, we identified the key variables that significantly influenced the electricity demand gap during the pandemic, particularly between 2020 and 2021, when the impact of the pandemic was most pronounced. It is also crucial to examine the pandemic's ongoing effects on electricity demand beyond 2021. Post-pandemic, the consumption patterns in commercial, industrial, and residential sectors have varied considerably, with some categories reverting to pre-pandemic routines and others undergoing substantial changes. As we move forward, forecasting future electricity demand becomes increasingly complex due to the lasting impacts of the pandemic. The methodology proposed in this study effectively analyzes the direct effect of key variables on the targeted demand. However, constructing a hierarchical structure for factors that may indirectly influence the demand gap could provide a more comprehensive understanding of the increasingly intricate demand dynamics observed in recent years [48]. Although sparse modeling is a widely utilized machine learning technique for analyzing important variables mechanically, supplementing model outputs with literature reviews and expert consultations is crucial for accurately discerning the physical relationships among variables. For example, investigating the relationships between changes in long-term electricity demand and ESG (environmental, social, and governance) factors helps clarify a physical interpretation that considers the growth of the country and the company [49,50]. Future research should also focus on developing forecasting methodologies that adequately account for the uncertainty associated with key variables. These variables can exhibit high volatility over time and may significantly impact demand gaps based on economic, lifestyle, and environmental conditions. Such an approach will be essential for accurately projecting electricity demand in a post-pandemic world, ensuring that energy systems are resilient and responsive to ongoing and future changes.

## Data availability statement

Data will be made available on request. The data used in this study is publicly accessible as open data. Table 2 in the article provides a summary of the sites where the data can be collected.

## Ethics declarations

Informed consent was not necessary for this study since the data utilized originated from public databases.

## Funding

The authors did not receive any funding for this article.

## CRediT authorship contribution statement

**Nanae Kaneko:** Writing – original draft, Visualization, Formal analysis, Data curation, Conceptualization. **Yu Fujimoto:** Writing – review & editing, Formal analysis, Conceptualization. **Hans-Arno Jacobsen:** Writing – review & editing. **Yasuhiro Hayashi:** Supervision.

## Declaration of competing interest

The authors declare that they have no known competing financial interests or personal relationships that could have appeared to influence the work reported in this paper.

## Figures and Tables

**Fig. A2:**
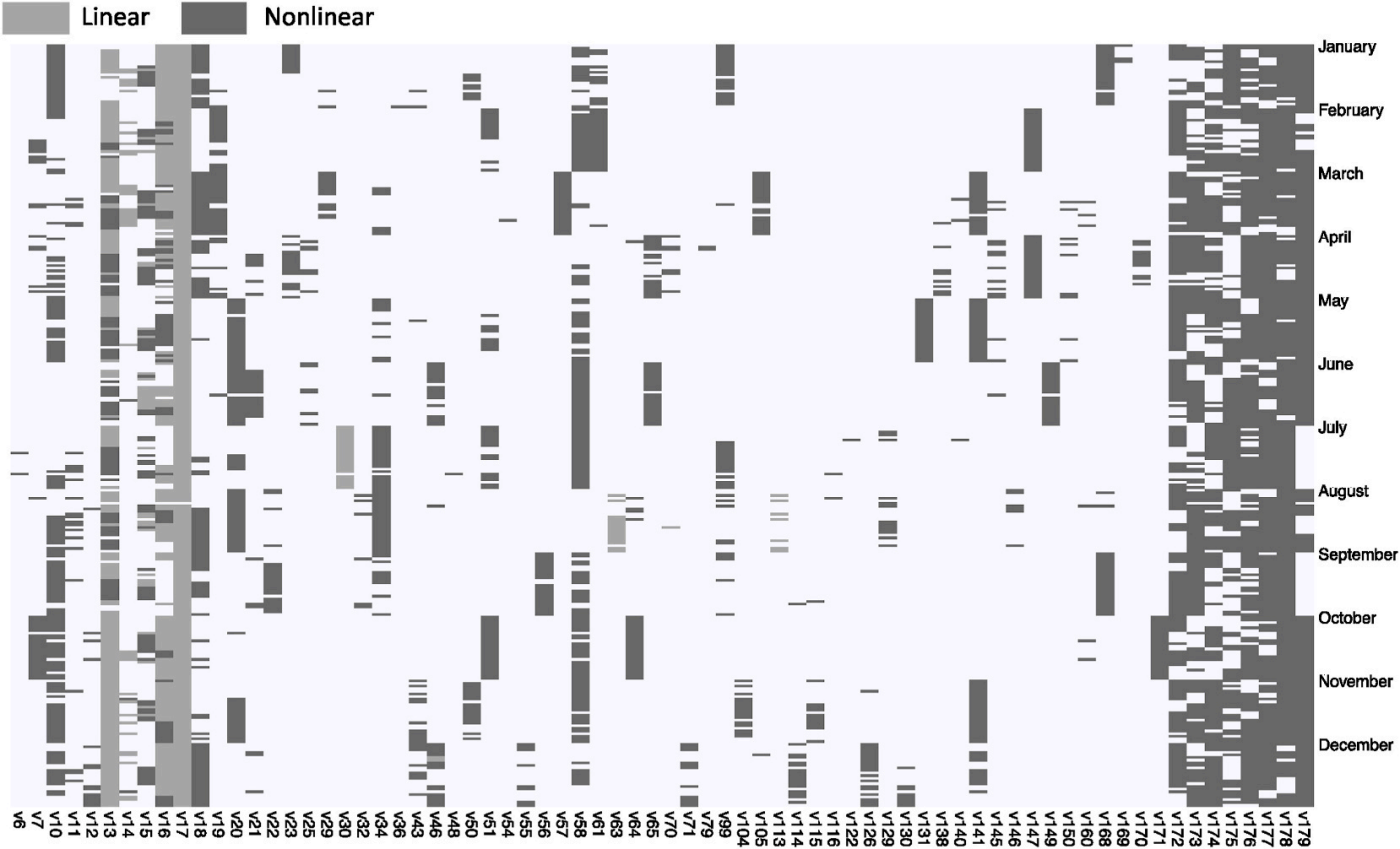
Selected variables derived by using PLAMs. The x-axis represents the selected variables, and the y-axis represents the transition of the situation (y,m,h).

**Table A1:** Details of all variables used in this study.

Category	Index	Item	Granularity
Installed generation capacity	1	"Biomass"	Yearly
	2	"Hydropower"	
	3	"Wind offshore"	
	4	"Wind onshore"	
	5	"Photovoltaics"	
	6	"Other renewable"	
	7	"Nuclear"	
	8	"Lignite"	
	9	"Hard coal"	
	10	"Fossil gas"	
	11	"Hydro pumped storage"	
	12	"Other conventional"	
Weather	13	"Humidity"	Hourly
	14	"Precipitation"	
	15	"Sun shine duration"	
	16	"Temperature"	
	17	"Wind speed"	
Stock index	18	"German Stock Index"	Daily
Production in industry	19	"Mining of coal and lignite"	Monthly
	20	"Extraction of crude petroleum and natural gas"	
	21	"Other mining and quarrying"	
	22	"Mining support service activities"	
	23	"Manufacture of food products"	
	24	"Manufacture of beverages"	
	25	"Manufacture of tobacco products"	
	26	"Manufacture of textiles"	
	27	"Manufacture of wearing apparel"	
	28	"Manufacture of leather and related products"	
	29	"Manufacture of wood and of products of wood and cork, except furniture; manufacture of articles of straw and plaiting materials"	
	30	"Manufacture of paper and paper products"	
	31	"Printing and reproduction of recorded media"	
	32	"Manufacture of coke and refined petroleum products"	
	33	"Manufacture of chemicals and chemical products"	
	34	"Manufacture of basic pharmaceutical products and pharmaceutical preparations"	
	35	"Manufacture of rubber and plastic products"	
	36	"Manufacture of other nonmetallic mineral products"	
	37	"Manufacture of basic metals"	
	38	"Manufacture of fabricated metal products, except machinery and equipment"	
	39	"Manufacture of computer, electronic and optical products"	
	40	"Manufacture of electrical equipment"	
	41	"Manufacture of machinery and equipment "	
	42	"Manufacture of motor vehicles, trailers and semitrailers"	
	43	"Manufacture of other transport equipment"	
	44	"Manufacture of furniture"	
	45	"Other manufacturing"	
	46	"Repair and installation of machinery and equipment"	
	47	"Electricity, gas, steam and air conditioning supply"	
Production in service	48	"Wholesale and retail trade and repair of motor vehicles and motorcycles"	Monthly
	49	"Wholesale trade, except of motor vehicles and motorcycles"	
	50	"Land transport and transport via pipelines"	

Category	Index	Item	Granularity
Production in service	51	"Water transport"	Monthly
	52	"Air transport"	
	53	"Warehousing and support activities for transportation"	
	54	"Postal and courier activities"	
	55	"Publishing activities"	
	56	"Motion picture, video and television program production, sound recording and music publishing activities"	
	57	"Programming and broadcasting activities"	
	58	"Telecommunications"	
	59	"Computer programming, consultancy and related activities"	
	60	"Information service activities"	
	61	"Legal and accounting activities"	
	62	"Architectural and engineering activities; technical testing and analysis"	
	63	"Advertizing and market research"	
	64	"Other professional, scientific and technical activities"	
	65	"Rental and leasing activities"	
	66	"Employment activities"	
	67	"Travel agency, tour operator and other reservation service and related activities"	
	68	"Security and investigation activities"	
	69	"Services to buildings and landscape activities"	
	70	"Office administrative, office support and other business support activities"	
Production in construction	71	"Buildings"	Monthly
	72	"Civil engineering works"	
GDP	73	"Gross Domestic Product(GDP)"	Monthly
Consumer price	74	"Bread and cereals"	Monthly
	75	"Meat"	
	76	"Fish and seafood"	
	77	"Milk, cheese and eggs"	
	78	"Oils and fats"	
	79	"Fruit"	
	80	"Vegetables"	
	81	"Sugar, jam, honey, chocolate and confectionery"	
	82	"Food products n.e.c."	
	83	"Coffee, tea and cocoa"	
	84	"Mineral waters, soft drinks, fruit and vegetable juices"	
	85	"Spirits"	
	86	"Wine"	
	87	"Beer"	
	88	"Clothing materials"	
	89	"Garments"	
	90	"Other articles of clothing and clothing accessories"	
	91	"Cleaning, repair and hire of clothing"	
	92	"Shoes and other footwear"	
	93	"Repair and hire of footwear"	
	94	"Actual rentals paid by tenants"	
	95	"Other actual rentals"	
	96	"Materials for the maintenance and repair of the dwelling"	
	97	"Services for the maintenance and repair of the dwelling"	
	98	"Water supply"	
	99	"Refuse collection"	
	100	"Sewerage collection"	

Category	Index	Item	Granularity
Consumer price	101	"Other services relating to the dwelling n.e.c."	Monthly
	102	"Electricity"	
	103	"Gas"	
	104	"Liquid fuels"	
	105	"Solid fuels"	
	106	"Heat energy"	
	107	"Furniture and furnishings"	
	108	"Carpets and other floor coverings"	
	109	"Repair of furniture, furnishings and floor coverings"	
	110	"Major household appliances whether electric or not"	
	111	"Small electric household appliances"	
	112	"Repair of household appliances"	
	113	"Major tools and equipment"	
	114	"Small tools and miscellaneous accessories"	
	115	"Nondurable household goods"	
	116	"Domestic services and household services"	
	117	"Pharmaceutical products"	
	118	"Other medical products"	
	119	"Therapeutic appliances and equipment"	
	120	"Medical services"	
	121	"Dental services"	
	122	"Paramedical services"	
	123	"Motor cars"	
	124	"Motor cycles"	
	125	"Bicycles"	
	126	"Spare parts and accessories for personal transport equipment"	
	127	"Fuels and lubricants for personal transport equipment"	
	128	"Maintenance and repair of personal transport equipment"	
	129	"Other services in respect of personal transport equipment"	
	130	"Passenger transport by railway"	
	131	"Passenger transport by road"	
	132	"Passenger transport by air"	
	133	"Passenger transport by sea and inland waterway"	
	134	"Combined passenger transport"	
	135	"Other purchased transport services"	
	136	"Telephone and telefax equipment"	
	137	"Telephone and telefax services"	
	138	"Equipment for the reception, recording and reproduction of sound and picture"	
	139	"Photographic and cinematographic equipment and optical instruments"	
	140	"Information processing equipment"	
	141	"Recording media"	
	142	"Repair of audio-visual, photographic and information processing equipment"	
	143	"Major durables for outdoor recreation"	
	144	"Musical instruments and major durables for indoor recreation"	
	145	"Maintenance and repair of other major durables for recreation and culture"	

Category	Index	Item	Granularity
Consumer price	146	"Games, toys and hobbies"	Monthly
	147	"Equipment for sport, camping and open-air recreation"	
	148	"Gardens, plants and flowers"	
	149	"Pets and related products"	
	150	"Veterinary and other services for pets"	
	151	"Recreational and sporting services"	
	152	"Cultural services"	
	153	"Books"	
	154	"Newspapers and periodicals"	
	155	"Miscellaneous printed matter"	
	156	"Stationery and drawing materials"	
	157	"Restaurants, cafes and the like"	
	158	"Canteens"	
	159	"Hairdressing salons and personal grooming establishments"	
	160	"Electrical appliances for personal care"	
	161	"Other appliances, articles and products for personal care"	
	162	"Jewelry, clocks and watches"	
	163	"Other personal effects"	
	164	"Insurance connected with the dwelling"	
	165	"Insurance connected with health"	
	166	"Insurance connected with transport"	
	167	"Other insurance"	
Google Trends	168	"Energy efficiency"	Monthly
	169	"Global warming"	
	170	"Renewable energy"	
	171	"Solar energy"	
Calendar	172	"Saturday(dummy)"	Daily
	173	"Sunday(dummy)"	
	174	"Monday(dummy)"	
	175	"Tuesday(dummy)"	
	176	"Wednesday(dummy)"	
	177	"Thursday(dummy)"	
	178	"Friday(dummy)"	
	179	"Holiday(dummy)"	

## Appendix

Figure A2 shows all variables selected for each seasonal situation.

The dataset utilized in this study is composed of 179 variables. These variables are sourced from open data, as cited in Table 2.

## Footnotes

1 The production index provides a measure of the price level or quantity of goods and services over time.
